# A strong, reversible, and conformal adhesive gel for diverse plants

**DOI:** 10.1126/sciadv.adz6379

**Published:** 2026-04-24

**Authors:** Jiayu Zhao, Zhecun Guan, Rohan Luthra, Patrick Opdensteinen, Brandon Ho, Xiao Li, Yong-Jin Park, Min Sub Kwak, Hyunhyub Ko, Nicole F. Steinmetz, Jinhye Bae

**Affiliations:** ^1^ Aiiso Yufeng Li Family Department of Chemical and Nano Engineering, University of California San Diego, La Jolla, CA 92093, USA.; ^2^ Shu and K.C. Chien and Peter Farrell Collaboratory, University of California, San Diego, La Jolla, CA 92093, USA.; ^3^ Center for Nano-ImmunoEngineering, University of California San Diego, La Jolla, CA 92093, USA.; ^4^ Department of Electrical and Computer Engineering, University of California San Diego, La Jolla, CA 92093, USA.; ^5^ Material Science and Engineering Program, University of California San Diego, La Jolla, CA 92093, USA.; ^6^ School of Energy and Chemical Engineering, Ulsan National Institute of Science and Technology (UNIST), Ulsan 44919, Republic of Korea.; ^7^ Institute for Materials Discovery and Design, University of California San Diego, La Jolla, CA 92093, USA.; ^8^ Department of Bioengineering, University of California San Diego, La Jolla, CA 92093, USA.; ^9^ Department of Radiology, University of California San Diego, La Jolla, CA 92093, USA.; ^10^ Moores Cancer Center, University of California San Diego, La Jolla, CA 92093, USA.; ^11^ Department of Chemical Engineering, Chung-Ang University, Seoul 06974, Republic of Korea.

## Abstract

Developing plant adhesives opens opportunities for studying and optimizing plant growth through precision pesticide and nutrient delivery, plant health monitoring, and human-plant interaction. However, diverse and changing topologies and chemical compositions of plants during growth present challenges in designing effective and universal adhesives. In this study, we address this challenge by developing a gel composite consisting of a biopolymer that enables dynamic covalent bonding with plant surfaces and cross-linked polyacrylamide, which provides adaptability. This composite gel demonstrates strong yet reversible adhesion on both hairy and nonhairy plant surfaces. Its adhesion strength exceeds that of previously reported noninvasive plant adhesives by an order of magnitude. This achievement enables localized and sustained drug delivery to plant tissues and allows for stable plant actuation via electrical stimulation, opening pathways for modulating sensitive plant systems.

## Teaser

A strong yet reversible adhesive gel enables cargo delivery and human-plant interaction via electrical stimulation.

## INTRODUCTION

Adhesives typically offer strong and durable bonding between surfaces and robust cohesive strength. However, ensuring compatibility with diverse substrates while maintaining an optimal balance between surface adhesion and bulk cohesion can be challenging (*1*). Over the past decades, adhesive technology has rapidly evolved to enhance bonding on both dry and wet surfaces (*2*–*4*). Classic adhesives such as epoxies (*5*), acrylates (*6*), and urethanes (*7*) have improved in durability, flexibility, and environmental resistance, meeting diverse industrial demands. Specifically, bioadhesives that are biocompatible and capable of forming strong adhesion to wet surfaces have opened the avenue for sensitive applications ranging from wound closure to implant fixation (*8*–*10*). Despite these recent advancements, the development of adhesives for plants has been largely overlooked.

Maintaining plant health is essential for agriculture, environmental sustainability, and food security. Effective interventions often rely on targeted delivery of bioactive compounds, such as nutrients, pesticides, or signaling molecules, to monitor plant physiology or confer protection (*11*, *12*). In parallel, plant-electronic interfaces have emerged as powerful platforms for monitoring electrophysiological signals for monitoring electrophysiological signals in real time, providing insights into plant responses and environmental interactions (*13*). However, the distinct challenges of plant surfaces, including their dynamic living nature and the presence of natural barriers such as waxy cuticles and hairy structures (i.e., trichomes) (*14*), have greatly hindered the development of effective bonding strategies. In particular, the wide variation in plant surface microstructure, presenting a major obstacle to creating universally effective plant adhesives (*15*, *16*). Structures such as microhooks or microneedles have been developed to facilitate the delivery of pesticides or nutrients to plants (*17*, *18*), featuring tips composed of nontoxic materials laden with the cargos. These structures are attached to the plant by penetrating the tissue, and cargos are released as the tip dissolves. However, this method causes damage to the plant tissue and could trigger adverse effects on plant health impacting crop yields. Luo *et al.* (*19*) have recently developed an adhesive gel for plant electrophysiology that transitions from sol to gel in response to temperature changes. This property enables the gel to be applied in liquid form, and it will then solidify, ensuring a nondestructive conformal contact on the hairy surfaces of plants. However, the adhesion strength of the thermogel is relatively low, with a maximum shear strength of 15 mN. This limitation stems from the inability of the thermogel to form chemical bonds with the plant surface and its weak mechanical properties, which leads to cohesive failure during shear tests, therefore limiting its practical use. As a result, there is a critical need for an adhesive that can form strong, noninvasive, and reversible bonding with plant tissues, particularly for applications in agriculture and botanical research (*20*–*22*).

**Fig. 1.**
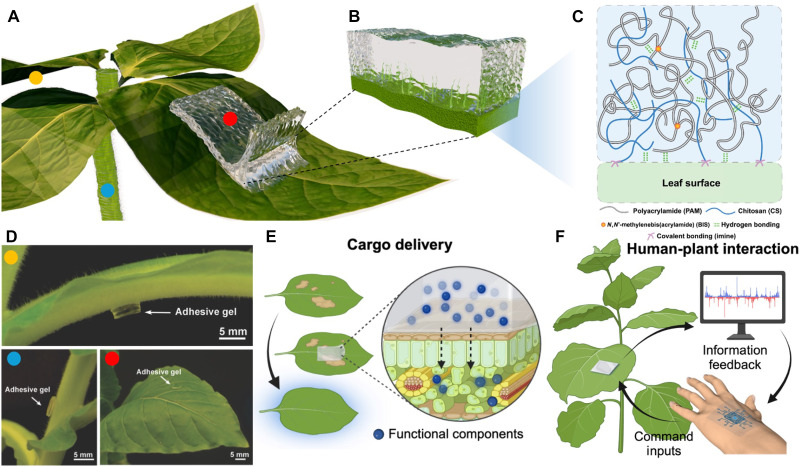
Overview of adhesive gel on hairy plants. (**A** and **B**) Schematics of the adhesive gel attached on hairy plant surface. (**C**) The adhesive gel is composed of an interpenetrated network with chemically cross-linked PAM entangled with high–molecular weight CS polymer chains. The adhesive gel can form conformal contact with the hairy plant surface owing to the carefully tuned mechanical properties and chemistry, creating reversible and strong adhesion via dynamic covalent bonding. (**D**) Photographs depict the adhesive gel adhering to the back stem (orange), middle stem (blue), and surface (red) of the hairy leaf. A high-magnification image reveals the adhesive gel forming seamless contact with the hairy leaf surface. The color coding corresponds to different locations illustrated in schematic (A). The adhesive gel enables (**E**) environmentally friendly cargo delivery system and (**F**) human-plant interaction. [(E) and (F)] Schematics were created in BioRender [Z. Guan (2025), https://BioRender.com/yyh2hzt].

In this work, we present a strategy for chemically and mechanically engineering gels to achieve strong yet reversible adhesion to both hairy and nonhairy plants by simply pressing the stand-alone adhesive gel onto the plant surface, allowing the formation of conformal contact between adhesive gel and surface of leaf (Fig. 1, A and B). The plant adhesive gel was synthesized by simultaneously polymerizing and cross-linking of the polyacrylamide (PAM) from a mixture of high–molecular weight chitosan (CS) and PAM precursor (see Materials and Methods). The amino groups on the CS polymer chain allow for the formation of dynamic covalent bonds with the ketone/aldehyde groups on the leaf surface (Fig. 1C), facilitating robust and reversible adhesion. As indicated visually, the gel not only adheres conformably to various types of plant surfaces (i.e., back stem, middle stem, and leaf surface) but also bears its own weight when attached to a plant stem (Fig. 1D). The mechanical properties of the cross-linked gels have been carefully tuned to ensure conformable contact with both hairy and nonhairy plant surfaces, using *Nicotiana benthamiana* (benth) and *Vigna unguiculata* (cowpea) as model plants with hairy and nonhairy surface characteristics, respectively. The synergistic effect of chemical and mechanical attributes achieves an optimal balance between surface adhesiveness and bulk cohesion, resulting in exceptional adhesion strength that represents the highest record (interfacial toughness, 87.2 ± 17.2 J/m^2^; adhesion strength, 10.1 ± 0.9 kPa) for a noninvasively applied plant adhesive. This innovative approach to developing gel adhesives for various plant surfaces could enable localized cargo delivery to plants (Fig. 1E), facilitating human-plant interaction and supporting the attachment of wearable sensors for plant health monitoring (Fig. 1F).

## RESULTS

An ideal plant adhesive should be mechanically tough to prevent cohesive failure, flexible to accommodate plant growth, transparent to facilitate normal chlorophyll function, capable of forming strong adhesion with diverse plant surfaces, and remain nontoxic to the plant. To fulfill these criteria, our adhesive gel has been engineered with an interpenetrating polymer network comprising two key components: a loosely cross-linked PAM for elasticity, and CS polymers as a source of amino groups, which react with the plant surface to establish dynamic imine bonds. The adhesive gel will be referred to as CS/PAM in the following discussion. While the major component across different species of plants is alkanes (> 50%), it is reported that rich aldehyde and ketone groups of 4 to 31% are also present in plenty of plants (*14*). The Fourier transform infrared (FTIR) results of benth and cowpea leaves further confirmed this composition (fig. S1A). The presence of aldehyde/ketone groups on the leaf surface allows the formation of imine bonds through Schiff’s base reaction upon the attachment of CS/PAM (fig. S1, B and C) (*23*, *24*). Because of the dynamic nature of this reaction, reversed attachment (i.e., detachment) can be achieved simply by adding water, where the imine group absorbs the water molecule and generates the aldehyde/ketone and amino groups again.

**Fig. 2.**
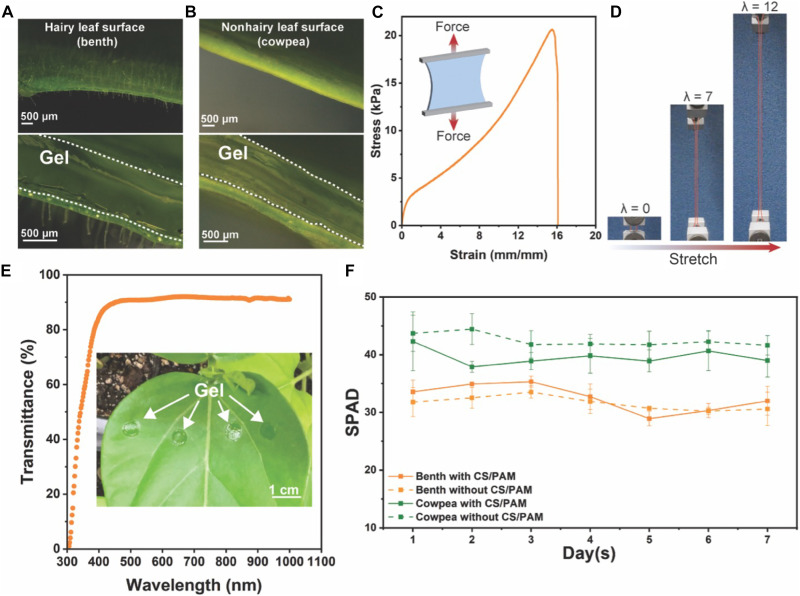
Mechanical and optical properties and biocompatibility of CS/PAM. (**A**) Hairy and (**B**) nonhairy leaf surface of benth and cowpea, respectively; CS/PAM form seamless contact with hairy and nonhairy leaf surface. (**C**) Strain-stress curve of CS/PAM and (**D**) images showing the stretched CS/PAM at various strains (λ) during the tensile test. (**E**) The light transmittance of CS/PAM in the wavelength range of 300 to 1000 nm. Inset photograph shows the transparency of the gels on a cowpea leaf. (**F**) Measurement of chlorophyll content, as represented by SPAD value, over 7 days for benth and cowpea leaves with and without the attachment of CS/PAM gel (experiment and control group, respectively). The error bars represent the SD.

It has been well recognized that a stretchable and tough gel favors a loosely cross-linked yet densely entangled network (*25*). Therefore, we mechanically engineered our gel by formulating it with a relatively high acrylamide (AM) monomer concentration (4 M) and low cross-linker [*N*,*N*′-methylenebisacrylamide (BIS)] amount (0.06 wt % relative to the weight of AM). A high–molecular weight CS was incorporated to further enhance the physical entanglement. The resulting CS/PAM exhibits excellent flexibility and compliance, allowing it to form conformal contact with benth (Fig. 2A) and cowpea (Fig. 2B), which represent hairy and nonhairy surfaces, respectively. Mechanical characterization of CS/PAM demonstrates a remarkable toughness of 197 ± 30 J/m^3^ and the ability to stretch to more than 17 times its original length (Fig. 2C). Figure 2D shows a visual representation to demonstrate its excellent stretchability. The CS/PAM also exhibits excellent transparency (~90% transmittance), ensuring the photosynthesis of the plants (Fig. 2E). To address the potential cytotoxic effect of the residual reagents on plant cells, we thoroughly purified the CS/PAM by washing it with tap water after curing. Chlorophyll content, widely acknowledged as one of the most important pigments in photosynthesis, serves as a key indicator of plant health (*26*). Therefore, we measured the chlorophyll content over 7 days in both benth and cowpea plants, with and without the application of CS/PAM. No substantial difference was observed between the experimental group (plants with CS/PAM) and the control group (plants without CS/PAM) for both plants (Fig. 2F), except for a temporary decrease in the soil-plant analysis development (SPAD) value of cowpea on day 2, which rebounded on day 3. Overall, all plants with the attachment of CS/PAM remain healthy after 7-day application with no noticeable physical difference compared with control groups, demonstrating the outstanding biocompatibility of the CS/PAM.

**Fig. 3.**
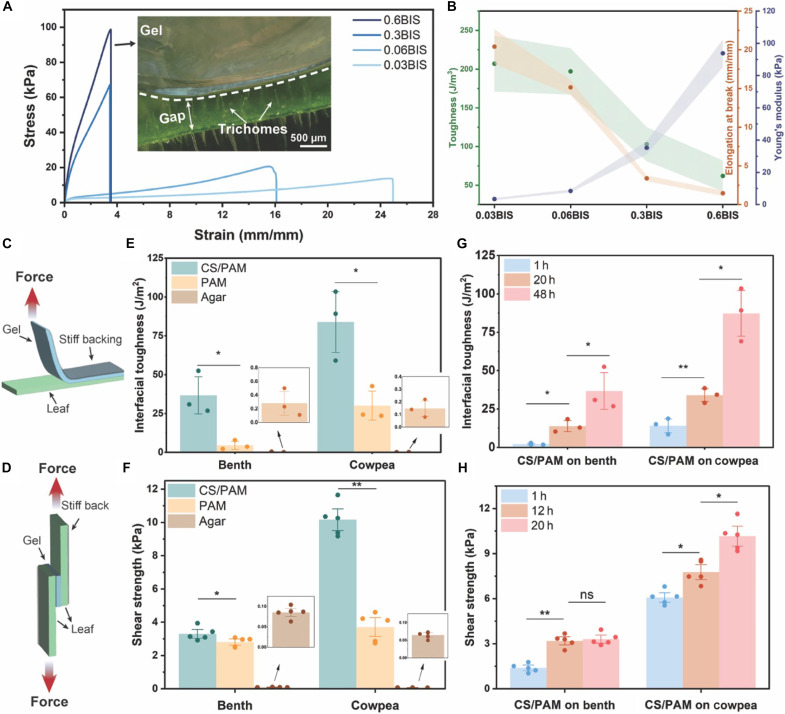
Effects of the mechanical properties of CS/PAM on gel-plant contact and the adhesion performance of the optimized CS/PAM. (**A**) Stress-strain curve and (**B**) mechanical properties of CS/PAM with different BIS concentrations. The inset image in (A) highlights the poor contact between the stiff CS/PAM and benth, resulting from the highest BIS concentrations. Schematics of (**C**) 90° peeling test and (**D**) lap shear test. Comparison of the (**E**) interfacial toughness and (**F**) shear strength of CS/PAM, PAM, and agar after 48 hours of application on benth and cowpea leave surfaces, respectively. The time-dependent (**G**) interfacial toughness and (**H**) shear strength of CS/PAM on benth and cowpea leave surfaces, respectively. The error bars represent the SD. Statistical analysis was performed by paired *t* test. *P < 0.05, **P < 0.01. ns, not significant.

To investigate the impact of mechanical properties of CS/PAM on adhesion to hairy plant surfaces, we varied the BIS concentration in the precursor solution from 0.03 to 0.6 wt % relative to the AM weight. The cured gels, designated as *x*BIS—where *x* represents the BIS concentrations 0.03, 0.06, 0.3, and 0.6 wt %—were tested for their stress-strain responses. Gels with 0.03BIS and 0.06BIS displayed initial elastic deformation (up to a strain of ~0.05 mm/mm), which then transitioned to plastic deformation until failure (Fig. 3A) (*27*). Their Young’s moduli were 3.39 ± 0.64 and 8.41 ± 0.84 kPa, with corresponding toughness of 207 ± 36 and 197 ± 30 J/m^3^ and elongations at break of 20.41 ± 2.25 and 15.14 ± 0.91 mm/mm, respectively (Fig. 3B). In contrast, gels with higher BIS concentrations of 0.3BIS and 0.6BIS exhibited purely elastic deformation until rupture, with significantly higher Young’s moduli of 35.17 ± 4.06 and 93.79 ± 8.63 kPa, lower toughness values of 103 ± 22 and 62 ± 20 J/m^3^, and reduced elongations at break of 3.38 ± 0.36 and 1.45 ± 0.35 mm/mm, respectively. These differences in their mechanical properties are originated from the polymer chain and network morphology. With a lower BIS concentration, the cross-linking density is relatively low, and the polymer chains between cross-linking points are longer than those with higher BIS concentrations. This results in a higher degree of chain mobility and entanglement. When stretched, the tension is transmitted along the chain and to numerous other chains through entanglements before any chain breakage occurs (*25*), endowing the gel with excellent flexibility and toughness.

The contact area is crucial in determining the adhesion properties of materials. Soft materials that can compel and conform to a surface typically exhibit stronger adhesion than rigid materials. This is because rigid materials tend to impose limited contact area and relatively low adhesive strength (*28*). Consequently, because of the high stiffness of gels with 0.6BIS and 0.3BIS, they demonstrate poor adhesion to the benth leaf surface and thus easily detach when subjected to external disturbance, such as airflow generated by a hair dryer (Dyson Supersonic, flow rate of 13 liters/s; movie S1). The intervening hairs (i.e., trichomes) hinder the contact with the leaf epidermis, as shown in the inset image of Fig. 3A. Notably, even when the gels with 0.6BIS and 0.3BIS are pressed onto the hairy leaf surface with an external force for ~1 min, they revert to their original state with a gap reappearing between the gel and the leaf’s epidermis once the force is removed. This is presumably because the gels’ elasticity suppresses adhesion under these conditions, where the relatively weak intermolecular forces (i.e., hydrogen bonding) that dominate adhesion in the initial stage of application (details discussed in Fig. 3) cannot overcome the elastic restoring force that the gel exhibits in its tendency to return to its original shape. In contrast, the gels with 0.03BIS and 0.06BIS show seamless contact with benth leaf epidermis despite the presence of hairs, maximizing the contact area (Fig. 2A). Therefore, strong and stable adhesion on benth leaf surface is created and maintained even under a vigorous airflow of 13 liters/s (movie S2). Although the gel with 0.03BIS exhibits a slightly higher toughness and elongation at break than that of 0.06BIS, the low Young’s modulus makes handling difficult. Therefore, gel with 0.06BIS concentration is selected for subsequent characterizations, hereafter referred to as CS/PAM.

To evaluate the adhesion performance of the CS/PAM, we measured the interfacial toughness by 90° peel tests and the shear strength by lap shear tests. The resulting adhesion performance was compared with that of PAM and agar. PAM serves as the control sample to evaluate the influence of CS on adhesion, while agar is commonly used to attach to plants for measuring their endogenous electrical signals (*29*, *30*). Benth and cowpea, our model plants, were used to evaluate the adhesion performance on hairy and nonhairy leaf surfaces, respectively. In the 90° peel test, a strip of gel is adhered to the surface of the target leaf (either benth or cowpea), where an upward force is applied to the stiff backing layer and peels the gel away from the leaf at a 90° angle (Fig. 3C and see details in Materials and Methods). In the lap shear test, the gel is sandwiched by two leaves, where one end of the leaf was fixed and the other end of the leaf was stretched vertically (Fig. 3D and see details in Materials and Methods). For both measurements, a thin and stiff backing layer (3M Scotch tape) was attached to either the surface of the gel or leaf to prevent their shape deformation along the peeling or shearing direction. The gels were attached to the leaf surfaces, followed by gentle pressing (~500 Pa) for 1 min, and were left for varied periods before testing. After 48 hours of attachment, the CS/PAM demonstrates robust adhesion on hairy and nonhairy leaf surfaces, exhibiting toughness values of 36.7 ± 13.8 and 87.2 ± 17.2 J/m^2^ and strength values of 3.3 ± 0.4 and 10.1 ± 0.9 kPa, respectively (Fig. 3, E and F), which are significantly higher than those of PAM alone. This difference aligns with our hypothesis: The primary interaction of PAM with leaf surfaces is limited to physical (hydrogen) bonding, while the addition of CS significantly enhances adhesion by initiating dynamic covalent bonds. This emphasizes the crucial role of CS in enhancing gel-to-leaf surface adhesion.

To investigate the temporal evolution of adhesion, we measured the interfacial toughness at 1, 20, and 48 hours and the shear strength at 1, 12, and 20 hours, following the application of CS/PAM on leaves. The maximum application time for the former case was set to 48 hours because the leaves become brittle and experience mechanical failure when the gel is being peeled off, making them unsuitable for the 90° peeling test for a longer period. Similarly, the latter case had its duration limited to 20 hours, as leaves invariably break first when subjected to stretching. Over time, the interfacial toughness and shear strength between CS/PAM and both benth and cowpea exhibit a significant upward trend. For the CS/PAM-benth interface, the toughness rises over 17-fold from 2.1 ± 0.6 to 36.7 ± 13.8 J/m^2^, while for the CS/PAM-cowpea interface, it increases over sixfold from 14.1 ± 5.2 to 87.2 ± 17.2 J/m^2^, as observed at time intervals of 1, 20, and 48 hours (Fig. 3G). Similarly, the shear strength at the CS/PAM-benth interface increased more than 2.5-fold from 1.3 ± 0.2 to 3.3 ± 0.4 kPa, and at the CS/PAM-cowpea interface, it increased over 1.6-fold from 6.1 ± 0.5 to 10.2 ± 0.9 kPa within 20 hours (Fig. 3H). To further explore the adhesion mechanism over time, we compared the adhesion obtained between CS/PAM and leaves with the one between PAM and leaves, respectively. For both cowpea and benth, the interfacial toughness values of CS/PAM and leaves are slightly higher than that of using PAM within the first hour but not statistically significant (fig. S2, A and B). However, after 20 hours, the interfacial toughness values at the interface of CS/PAM-cowpea and CS/PAM-benth are 2.3 and 3.8 times higher than the ones using PAM, respectively. This deviation continues to increase after 48 hours, where the interfacial toughness values at the interface of CS/PAM-cowpea and CS/PAM-benth are more than 2.8 and 8 times higher than the ones using PAM, respectively. In contrast to the continuously increased interfacial toughness between CS/PAM and leaves over time, the ones using PAM show little increase across the 48-hour testing period. This result matches with our hypothesis that it is hydrogen bonding primarily contributes to the adhesion between PAM and leaves, which is unlikely to change over time. By comparing the results obtained using CS/PAM and PAM, we can conclude that hydrogen bonding dominates the system for promoting the adhesion between gels and leaves within the first hour. However, more CS polymer chains can gradually diffuse to the leaf surfaces, thus establishing more dynamic covalent bonds by forming imine as time passes (*31*). Note that this reaction can form water molecules, which can be preferentially absorbed by the hydrophilic PAM network, driving Schiff’s base reaction forward by removing the water molecules at the interface (*32*). Therefore, the adhesion between CS/PAM and leaves enhances over time.

Furthermore, four commercially available plant species were examined to demonstrate the general applicability of this gel adhesive across diverse plant surfaces, including two dicots—*Ocimum basilicum* (basil) and *Euphorbia milii* (crown-of-thorns)—and two monocots—*Epipremnum aureum* (pothos) and *Zantedeschia aethiopica* (calla lily). Adhesive performance on the adaxial leaf surfaces was assessed using both 90° peel test and lap shear tests to provide a comprehensive characterization of gel attachment (fig. S3). All tested leaves have shown strong adhesion with a shear strength of >5 kPa and a toughness of >27 J/m^2^, indicating strong adhesion between the gels and plant surfaces.

**Fig. 4.**
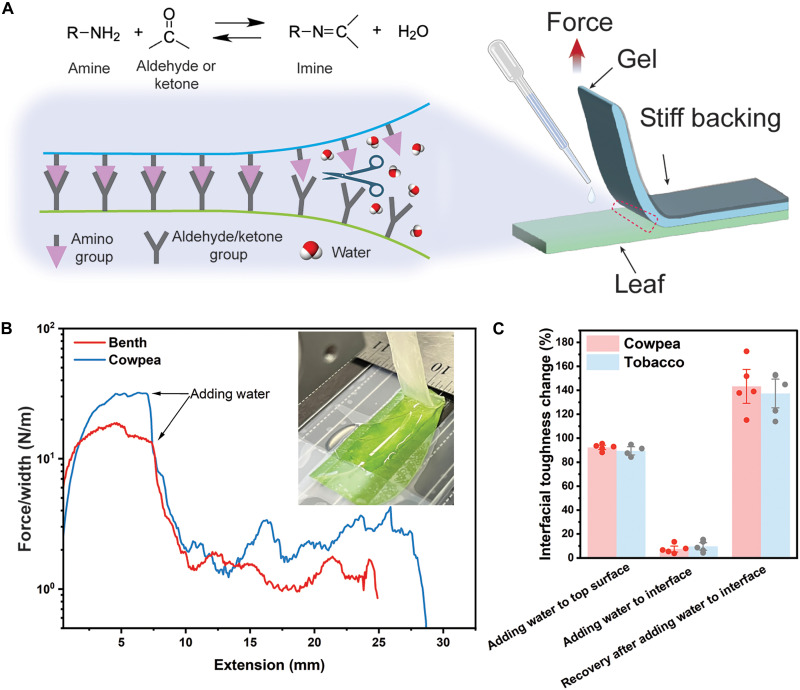
Reversible adhesion between adhesive gels and leaf surface. (**A**) Schematic depicting the on-demand loss of adhesion by adding water, in which imine can hydrate and revert to the original amino group and aldehyde/ketone. (**B**) Curves of the peeling force per width of CS/PAM as a function of extension for CS/PAM on cowpea and benth leaf surfaces, respectively. Water was added during the 90° peeling test (inset image), where a large drop of adhesive strength occurred. (**C**) Comparison of the interfacial toughness of CS/PAM on cowpea and benth after adding water at the gel-leaf interface and recovery, as well as after adding water on the top of the gel. The interfacial toughness restoration of CS/PAM on cowpea and benth leaves involves reattaching the peeled-off CS/PAM after removing excess water, pressing gently (~500 Pa) for 1 min, and resting for 48 hours before retesting with 90° peeling. A control experiment was performed by adding water to the top surface of the gel. The error bars represent the SD.

In addition to strong adhesion, reversibility in plant adhesives offers substantial benefits, including the ability to attach and detach materials to plant surfaces without damage, making it ideal for nondestructive applications such as nutrient/cargo delivery for plants or attaching sensors for monitoring plant health. The dynamic nature of Schiff’s base reactions not only imparts the CS/PAM-leaf system with time-dependent adhesion behaviors but also enables reversible adhesion simply by adding water. Imine hydrolysis occurs when water molecules are introduced to the system, resulting in the formation of the corresponding amino group and a carbonyl-containing compound (either an aldehyde or a ketone) (*33*). To confirm this, we transferred 600 μl of deionized (DI) water to the CS/PAM and leaf interface during a 90° peeling test (Fig. 4A). For both benth and cowpea leaves, the force per width decreased by an order of magnitude upon water addition (Fig. 4B). Consequently, the adhesion was significantly reduced, as evidenced by the interfacial toughness changes, with those tested on CS/PAM-benth and CS/PAM-cowpea being less than 10% of their initial values (i.e., before water was added). Because of the reversibility of Schiff’s base reaction, the adhesion was restored by reattaching the CS/PAM onto the leaf surfaces. After removing the excessive water from leaf surfaces using Kimwipes, the CS/PAM was gently pressed (~500 Pa) onto the leaves for 1 min. After 48 hours, the adhesion fully recovered (Fig. 4C). While significant leaf drying after 48 hours restricted the recovery test to 1 cycle, the results effectively demonstrate that the plant adhesive developed in this study is capable of forming reversible bonding. To further validate this mechanism, we performed a control experiment by applying the same amount of water to the top surface of the gel. This resulted in only a marginal reduction in interfacial toughness, decreasing by 10.5% for benth and 7.8% for cowpea relative to the dry condition. These reductions are substantially smaller than the significant loss of adhesion observed when water is introduced directly at the gel-leaf interface. In addition, to assess the reusability of the gel, we first adhered it to a benth leaf that remained on the plant for 48 hours. For subsequent cycles, we used freshly excised leaves for each reattachment step to minimize the influence of leaf dehydration (fig. S4). Despite undergoing dehydration over 48 hours, the gels exhibited no significant change in Young’s moduli. The interfacial toughness remained in the same range for the first two attachment cycles and then gradually declined in later cycles. Overall, the gel maintained effective adhesion through for up to four reuse cycles.

To address the concern that water-triggered imine hydrolysis may compromise gel adhesion under natural outdoor conditions, we evaluated the stability of gel adhesion under simulated rainfall conditions. The mist of drizzle was mimicked using a spray bottle, and the violent rain was simulated with a showerhead. Four plant species were tested under the drizzle setting for 1 min, and two were tested under the violent rainfall setting for 20 min. The simulated rainfall depth for the heavy rain condition reached 2324.2 mm, corresponding to ~46.5 hours of continuous violent rainfall at 50 mm/hour, the threshold defined by MANOBS (Manual of Surface Weather Observations). In all cases, the gels remained firmly attached to plant surfaces, demonstrating robust rainfall resistance (fig. S5). To evaluate adhesion robustness under water exposure, we quantified the interfacial toughness of the adhesive gel under simulated drizzle and heavy rain conditions (fig. S6). While the measured toughness decreased substantially because of partial gel swelling and the decreasing adhesion at the interface with the stiff backing, adhesion remained sufficiently strong to maintain stable attachment to leaf surfaces. A detailed comparison of this work with previously reported approaches (*19*, *34*–*38*) is provided in table S1, which compiles other noninvasive methodologies alongside our system. To the best of our knowledge, this is the first report of a nondestructive plant adhesive capable of forming both strong and reversible adhesion simultaneously. The reversible nature of this adhesion, controlled by the presence of water, simplifies field applications and minimizes environmental impact, qualifying it as a sustainable and cost-effective candidate in materials science with a wide range of potential applications.

**Fig. 5.**
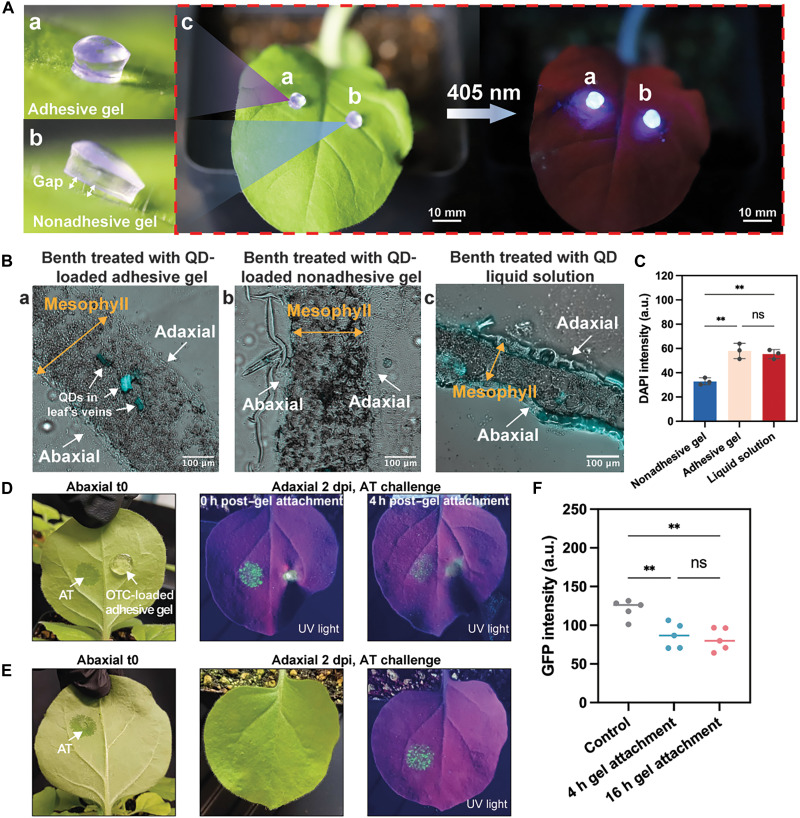
Cargo delivery to plants and in planta antibacterial activity of antibiotic-containing adhesive gels. (**A**) Photographs showing the attachment of QD-loaded (a) adhesive gel and (b) nonadhesive gel on benth leaf surfaces. The photoluminescence excited by 405-nm wavelength light indicated that the QDs were successfully loaded into both gels. Adhesive and nonadhesive gel formulated as CS/PAM with 0.06 and 0.6 wt % BIS, respectively. (**B**) Representative fluorescent microscope images of cryosectioned benth leaves after being treated with (a) QD-loaded adhesive gel, (b) QD-loaded nonadhesive gel, and (c) QD liquid solution for 4 hours, merged with bright-field and DAPI channels (false coloring). (**C**) Corresponding fluorescence signal intensity of QDs in benth cryosections. Values were derived from fluorescent images shown in figs. S5 to S7 using ImageJ. a.u., arbitrary units. (**D**) Protection against bacterial infection. Benth plants (*n* = 3) were challenged with transgenic *A. tumefaciens* (pJL-TRBO-GFP) (AT) via syringe infiltration 0 or 4 hours after attaching gels containing oxytetracycline (OTC; 1.25 mg/ml). The transgenic agrobacteria express green fluorescent protein (GFP) upon successful infection, which was visualized under ultraviolet (UV) light 2 days post–agrobacterium infection (2 dpi) [left: AT; right: OTC-loaded adhesive gel at both t0 and 2 dpi for abaxial and adaxial surfaces]. (**E**) Agrobacterium infection control. Untreated benth (*n* = 3) was challenged with transgenic *A. tumefaciens* (pJL-TRBO-GFP) via syringe infiltration. Expression of GFP was visualized under UV light after 2 dpi. (**F**) Fluorescence intensity of GFP in leaves measured at 2 dpi, following agrobacterial challenge 0, 4, or 16 hours after attaching OTC-loaded gels. Values were derived from fluorescent images using ImageJ. The error bars represent the SD. Statistical analysis was performed by one-way analysis of variance (ANOVA) with a post hoc Tukey’s test. **P < 0.01.

Here, we showcase the cargo delivery system as one of the potential applications. Unlike conventional foliar spray methods that deploy cargos such as pesticides and nutrients onto crops, encapsulating these substances in gels allows for controlled and sustained release to the target plant tissue. This method enhances effectiveness and minimizes environmental impact by reducing runoff. In addition, it lowers toxicity risks to nontarget organisms, including humans. To prove the concept, we prepared CS/PAM gels loaded with quantum dots (QDs) (refer to Materials and Methods for details) and attached them to the adaxial surface of a benth leaf (Fig. 5A). QDs are selected as the representative cargo because they are fluorescent, nanometer-sized particles that are taken up and translocated by plants through cell wall pores (7 to 20 nm) (*39*–*41*). Their fluorescent characteristics enable visualization within plant tissue under fluorescence microscope. We investigated the effect of gel adhesiveness in cargo delivery on hairy plant (benth), using CS/PAM with 0.06 wt % BIS as the adhesive (Fig. 5Aa) and CS/PAM with 0.6 wt % BIS as the nonadhesive variant (Fig. 5Ab). QDs were loaded to the adhesive and nonadhesive gels, respectively, as indicated by the fluorescence upon irradiated by 405-nm wavelength light (Fig. 5Ac). After 4 hours of applying the QD-loaded gels and QD liquid solution to the benth leaf surface, we removed the gels and rinsed the leaf surfaces with tap water to wash away any remaining QDs. The treated areas were cryosectioned and examined using Nikon Eclipse Ni fluorescence microscope. Notably, strong QD signals were detected in the veins of benth leaves treated with QD-loaded adhesive gel (Fig. 5Ba and fig. S7), whereas those treated with QD-loaded nonadhesive gel showed only weak signals near the epidermis (Fig. 5Bb and fig. S8). 4′,6-Diamidino-2-phenylindole (DAPI) signals in QD liquid solution treated leaf cryosections demonstrate larger fluorescent regions that are mostly around the cuticle and epidermis (Fig. 5Bc and fig. S9). Compared with liquid application, gel-enabled delivery prolongs leaf contact and sustained exposure of QDs, enhancing internalization and producing a more effective vein-localized accumulation (*42*). In addition, QDs delivered by adhesive gel present an average fluorescence signal intensity 1.87 times higher than those delivered by nonadhesive gel (Fig. 5C). This increase can be attributed to the adhesive gel’s seamless contact with the hairy plant surface, which facilitates QD transportation through the leaf stomata and vein, while QD signals remain at mesophyll when transported by nonadhesive gels (*43*). These findings underscore the relevance of the gel-enabled QD delivery approach for achieving effective internal distribution of functional compounds in plants. To further investigate the QD diffusion speed in plant tissue using adhesive gel, nonadhesive gel, and a QD liquid solution, we used a real-time imaging method that records a series of images at a 20-min interval over 4 hours (fig. S10). The delivery speed is calculated by dividing the diffusion distance by the diffusion time. As expected, the QD delivery using the QD solution is most rapid, at 67.6 ± 11.1 μm/hour, while delivery rates using adhesive and nonadhesive gels are 31.9 ± 3.9 and 13.8 ± 3.1 μm/hour, respectively (movies S9 to S11).

Building on the proof-of-concept QD delivery studies, we next sought to demonstrate an application and therefore used the gel adhesives to deliver antibiotics to plants to treat and manage plant infections. Here, we used the broad-spectrum antibiotic oxytetracycline (OTC) as a model for targeted delivery (*44*) and transgenic *Agrobacterium tumefaciens* (pJL-TRBO-GFP), a common plant pathogen as a model for studying bacterial infections (*45*). Transgenic *A. tumefaciens* was chosen as a model to facilitate the detection of infection events through expression of green fluorescent protein (GFP) in plants. First, we tested the tolerance of benth for OTC and loaded adhesive gels with OTC (1.25 or 2.5 mg/ml); gels were attached to the abaxial surfaces of benth leaves (fig. S11, A to C). Our reasoning was that the thicker cuticle on the adaxial surface would impair antibiotic uptake. Notably, tissue damage was observed in leaves treated with gels containing higher OTC concentration (2.5 mg/ml), indicating a tolerance threshold for antibiotic dosage in plants. The minimal damage observed in leaf treated with OTC (1.25 mg/ml), however, did not affect overall plant growth as demonstrated by leaves observed 7 days postattachment (fig. S11D).

We therefore continued with an antibiotic concentration of 1.25 mg/ml for subsequent experiments. Nevertheless, the observed toxicity indicated that OTC diffused from the adhesive gels into the plant tissue as anticipated. Next, we tested the targeted protection from bacterial infections by challenging plants with transgenic *A. tumefaciens* via syringe infiltration 0, 4 or 16 hours after attaching OTC-loaded gels. Our hypothesis was that inhibitory OTC concentrations would be reached in plant tissues several hours after attaching OTC-loaded gels. Infection success or prevention thereof was monitored by GFP fluorescence under ultraviolet (UV) light at 2 days postinfiltration (Fig. 5, D and E). Compared to untreated controls, leaves treated with OTC (1.25 mg/ml)–loaded gels exhibited substantially reduced GFP signals, indicating that the OTC-loaded gels interfered with bacterial infections 4 hours after attaching the gels. Longer incubation times did not significantly enhance the protective effect (Fig. 5F). These results demonstrate that adhesive gels can serve as a localized antibiotic delivery platform, providing targeted protection while maintaining plant integrity.

To ensure the adhesive gel remains effective over time, particularly under field-relevant conditions, gels can be coated with a thin polydimethylsiloxane-benzophenone (PDMS-BP) layer to reduce dehydration and sustain adhesion (*46*). The PDMS-BP–coated gels exhibited a seamless interface between the hydrophobic PDMS-BP elastomer and the adhesive gel, ensuring robust interfacial adhesion and minimizing water loss (fig. S12A). Over a 7-day period, the coated gels exhibited significantly higher mass retention compared to bare gels under both laboratory and outdoor conditions. This demonstrates the effectiveness of the protective layer in sustaining adhesion and conformability to surfaces (fig. S12, B to D). Consequently, the gel adhesive can maintain conformal contact with plant surfaces throughout the tested duration. For fast-growing species such as *Arabidopsis thaliana* and *Helianthus annuus*, sustaining adhesion for even a few days to a week covers a significant developmental window of leaf growth (*47*–*49*). Therefore, the adhesive gel has emerged as a promising candidate for a noninvasive, precision drug delivery system in agricultural applications. It would facilitate the controlled and targeted delivery of agrochemicals through a slower diffusion rate than the liquid form, which is pivotal for enhancing crop management practices.

**Fig. 6.**
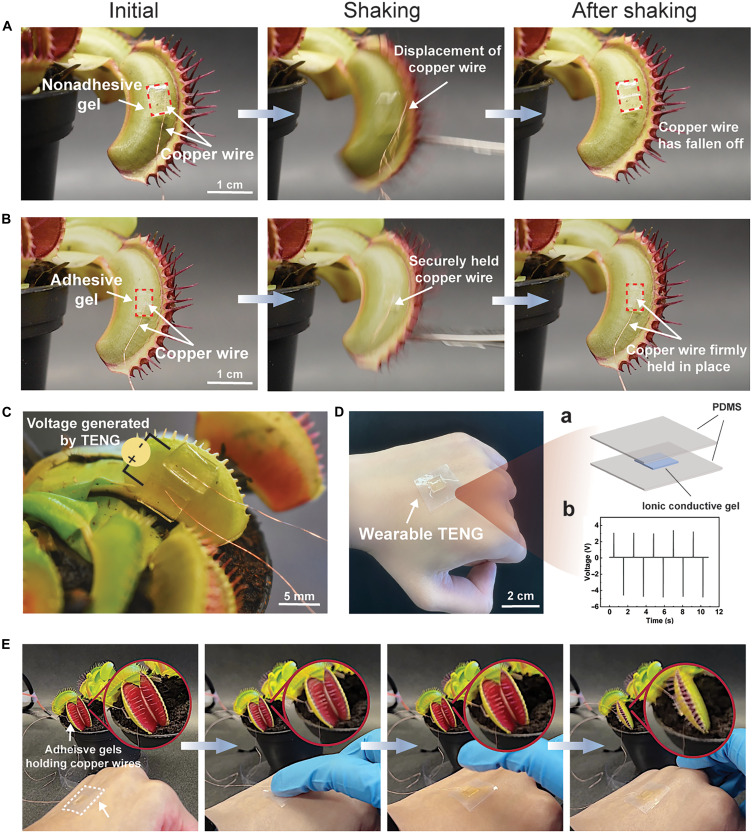
Stable human-plant interaction enabled by adhesive gels. (**A**) Nonadhesive and (**B**) adhesive gel holding copper wire on Venus flytrap before and after shaking. (**C**) The top view of the adhesive gels holding the copper wires that deliver the voltage generated by triboelectric nanogenerator (TENG). (**D**) Photograph of the wearable TENG attached to the skin. (a) The TENG comprised a layered structure where an ionic conductive gel (PAM-LiCl) is sandwiched by two BP-doped PDMS layers. (b) Voltage generated upon pressing and releasing the TENG (data obtained using a pressing pressure of 49 kPa and a contact speed of 1 Hz. (**E**) Actuation of the Venus flytrap by pressing and releasing the wearable TENG on the skin, generating electrical voltage that sends to the conductive adhesive gel on the Venus flytrap through conductive copper wire.

To extend the potential of adhesive gels, we advance the plant-human interface using the gel as a medium for interaction (*50*). Plants, such as the sensitive *Mimosa pudica* and Venus flytrap, transmit electrical signals within their structure in response to stimuli (*51*, *52*). The former folds its leaves upon touching, while the latter closes its lobe when its upper epidermis is touched. It is reported that a plant-based actuator using a Venus flytrap and a conformal electrode, consisting of a gold nanomesh sandwiched between an elastomer layer and a gel layer, could be wirelessly controlled via a smartphone to close the trap through electrostimulation (*38*). Representing a pioneering approach to plant-based actuators, this work lacks the discussion of gel adhesion to the Venus flytrap’s surface for reliable actuation, especially under disturbance, such as plant shaking. The following demonstration we show in this work aims to bridge this gap by evaluating the performance of adhesive versus nonadhesive gels in electrostimulation applications. The adhesive and nonadhesive gels were applied to the lobes of the Venus flytrap to secure the location of copper wires that delivered electric signals. We then shook the corresponding lobe to determine whether the copper wires could be maintained by the gels under conditions of disturbance. The nonadhesive gels, formulated as previously described, struggle to maintain attachment to the lobe’s surface and detach upon placement (movie S12). The poor conformability of the nonadhesive gel creates a gap between the gel and the lobe surface, resulting in a tenuous grip on the copper wire (fig. S13A), which leads to wire displacement with gentle shaking (Fig. 6A and movie S12). In contrast, the adhesive gels facilitate a robust connection to the Venus flytrap (fig. S13B), ensuring secure copper wire attachment even after several instances of vigorous shaking (Fig. 6B and movie S13). For comparison, this adhesive gel further surpasses three representative commercially available adhesives (3M magic tape, 3M delicate surface tape, and EZlifego double-sided tape) on both benth and cowpea leaves by offering the strong yet reversible adhesion that conforms to plant surfaces while allowing clean detachment without damaging the plants (fig. S14).

To demonstrate the augmented plant as sensor for human motion facilitated by the adhesive gels, we firmly held copper wires using a pair of adhesive gels on one of the Venus flytrap lobes (Fig. 6C). The copper wires deliver the voltage generated by a wearable triboelectric nanogenerator (TENG; Fig. 6D) that composed of a layered structure, where an ionic conductive gel (PAM-LiCl; see Materials and Methods for details) is sandwiched by the PDMS layers (Fig. 6Da and see Materials and Methods for details). Electrical properties of PAM-LiCl, CS/PAM, and CS/PAM-CaCl_2_ are presented in fig. S15. PAM-LiCl reaches the highest conductivity of ~2 S/m, and the inclusion of CaCl_2_ enhances the ionic conductivity of CS/PAM gel, improving its performance as a conformal electrode while maintaining adhesion. The working mechanism of TENG has been thoroughly studied elsewhere, and it is not the focus of this study (*53*–*55*), but, briefly, when a tribo-positive object (i.e., a finger) contacts the tribo-negative PDMS film of the TENG, opposite charges are generated on their surfaces (fig. S16). As these surfaces separate, static charges on the PDMS induce ion movement in the ionic gel, creating an excess ion layer. Electrons flow through external circuits to balance these charges. Reversing this movement, by bringing the dielectric film back toward the elastomer, reverses the electron flow. This repeated contact-separation cycle between the tribo-positive object and the TENG produces alternating current. The TENG we fabricated here exhibits a maximum voltage of 4.8 V using aluminum as the tribo-positive object (Fig. 6Db), which is higher than the electrostimulation threshold for the Venus flytrap (~1.5 V) previously reported (*38*). A signal module was used to transform the spike-like alternating voltage generated by TENG to ~3.2 V square waveform voltage so that the plant can recognize the signal better (fig. S17, A and B, and see Materials and Methods for details) (*56*). By pressing the wearable TENG on hand, one can close the lobe of the Venus flytrap without physically touching it, as the adhesive gel steadily holds the copper wire (Fig. 6E and movie S14). To verify that the actuation of the Venus flytrap is originated from the electrical signal rather than any mechanical disturbance generated when pressing the TENG device, we conducted a control experiment using nonconductive silicone tube to replace conductive copper wire. As expected, despite the voltage generated by pressing the TENG, no actuation of the Venus flytrap was observed (fig. S18 and movie S15).

## DISCUSSION

We have developed a strategy for chemically and mechanically engineering a gel that enables strong, yet reversible, adhesion to both hairy and nonhairy plant surfaces. The adhesive gel, consisting of a CS/PAM interpenetrating network, has been formulated to be flexible and stretchable, thus ensuring conformity to plant surfaces for a long growth period, including those with trichomes. The amino groups of CS polymers form dynamic covalent imine bonds with the ketone/aldehyde groups on leaf surfaces, leading to strong interfacial toughness values of 36.7 ± 13.8 and 87.2 ± 17.2 J/m^2^ with benth and cowpea, respectively, surpassing those of existing gel-based plant adhesives. To demonstrate its future applications, we used the adhesive gel for the cargo delivery system on plant tissues, achieving targeted, environmentally friendly release profiles superior to those of solution-based methods. When integrated with functional devices such as TENG, the adhesive gel facilitates human-plant interaction, as shown by a Venus flytrap closing its lobes in response to human motion without direct contact. This biocompatible, robust, reversible, and noninvasive adhesive opens avenues for various applications in plant engineering. For instance, pairing the adhesive with plant sensors and machine learning algorithms allows continuous, nonintrusive plant health monitoring for early disease detection (*57*). In addition, by embedding salt ions into the gel matrix, it can function as a noninvasive and stable morphable electrode for plant electrophysiology studies.

## MATERIALS AND METHODS

All chemicals were purchased from Sigma-Aldrich unless otherwise mentioned and used as received without further purification. To prepare the gels, we used CS (310 to 375 kDa, deacetylation ≥ 75%), AM, BIS, Irgacure 2959, and acetic acid. To prepare the salt solution, we used calcium chloride hydrate and lithium chloride. To make the PDMS molds, we used Ecoflex 00-30 (smooth-on). To prepare the PDMS layer of the TENG, we used Sylgard 184 (DOW) and BP. Water-soluble cadmium selenide zinc sulfide (CdSe/ZnS) QDs were purchased from NNCrystal US Corporation (carboxylic acid stabilizing ligands, 7- to 12-nm particle size, and emission peaks of 460 ± 10 nm) and used without further modifications. OTC hydrochloride was used to treat the plant pathogen. Venus flytrap, basil, crown-of-thorns, pothos, and calla lily were obtained from a grocery store.

### Preparation of CS stock solution

Acetic acid was used to prepare the acidic buffer solution. One milliliter of acetic acid was dissolved in 100 ml of DI water, followed by the addition of 2 g of CS powder. The mixture was vigorously mixed using an electric stirrer mixer (Mophorn) until the CS was completely dissolved.

### Preparation of gels

To prepare the PAM gel, 2.84 g of AM monomer, 1.7 mg of BIS (0.06 wt % with regard to AM), and 0.04 g of Irgacure 2959 were dissolved in 10 ml of DI water. After mixing, the mixture was injected into the PDMS mold with the desired dimension and cured under UV light at an intensity of 60 mW/cm^2^ for 10 min using Omicure (365 nm). Similarly, the CS/PAM gel was prepared using the same method as the PAM, except replacing the DI water with CS solution, and different BIS concentrations (0.03, 0.06, 0.3, and 0.6 wt % with regard to AM) were used to investigate how the mechanical properties influenced the adhesion properties. The as-prepared gels were carefully removed from PDMS mold using a carbon fiber tip tweezer, and the liner of adhesive transfer tape (3M) was used to facilitate the gel transferring. CS/PAM-CaCl_2_ as the conformal electrode for the plant is prepared using CS/PAM with 0.06 wt % BIS, and the cured gels were immersed in 0.1 M CaCl_2_ solution for 12 hours to endow ionic conductivity. Airflow was used to dry the gel surface before applying it to the plant.

### Preparation of the QD-loaded gels

The QDs were loaded into the gel matrix through a diffusion-driven method. Briefly, a rectangular gel of CS/PAM with a dimension of 10 mm by 30 mm by 2 mm was first prepared with the procedures mentioned above. For the adhesive and nonadhesive CS/PAM, we used formulations with 0.06 and 0.6 wt % BIS, respectively. After purification, they were biopsy-punched to disks with a radius of 5 mm. Then, they were left at room temperature for 24 hours to dry, followed by immersed in the 400 μl of QD solution (1 mg/ml) for 12 hours to reach the equilibrium state. For PAM gels with different BIS concentrations, similar treatment was performed before applying them to benth leaves.

### Gel dehydration and mass retention

For future applications, the gels can be coated with a PDMS-BP layer to avoid dehydration. The PDMS precursor was prepared by mixing the Sylgard 184 base, curing agents, and BP (10:1:0.18 by weight) using a Thinky mixer (THINKY USA, AR-100). The BP creates reactive sites on PDMS polymer chains, allowing for covalent bonding with the gel upon UV irradiation (*54*). The PDMS-BP layer was cured at 90°C for 1 hour, followed by the addition and polymerization of the gel precursor under UV light. Both bare gels and PDMS-BP–coated gels were adhered to glass slides and weighed every 24 hours under laboratory [~20°C, relative humidity (RH) of ~60%, and light intensity of ~500 lux for 8 hours/day)] and outdoor (direct sunlight peaks at ~150,000 lux; temperature and humidity data retrieved from a publicly available meteorological data) conditions. Indoor temperature and humidity were monitored with a hygrometer (ThermoPro), and light intensity was recorded using a lux meter (Smart Sensor). The mass fraction of the adhesive gel was calculated by subtracting the mass contributions of the glass slide and PDMS-BP layer from the total measured mass

$$\text{Mass fraction}=\frac{m_{\text{gel}},\text{Day}\;X}{m_{\text{gel}},\text{Day}\;0}=\frac{m_{\text{overall}},\text{Day}\;X-m_{\text{glass}}-m_{\text{PDMS}-\text{BP}}}{m_{\text{overall}},\text{Day}\;0-m_{\text{glass}}-m_{\text{PDMS}-\text{BP}}}$$

where $m_{\text{overall}}$ is the measured mass of the entire sample (PDMS-BP–coated gel on glass slide), $m_{\text{glass}}$ and $m_{\text{PDMS}-\text{BP}}\;$are constant throughout the measurement period, and $m_{\text{gel}}$ represents the calculated gel mass at each time point.

### Fabrication of TENG

Fabrication of TENG is adapted from a previous study. The PDMS-BP film was prepared by spin-coating the mixture of PDMS precursor, followed by a 90°C treatment for 1 hour, and the resulting thickness is around 140 μm. The film was then cut into 2 cm–by–2 cm square sheets using a laser cutter (Epilog Fusion Pro). The PAM-LiCl precursor is prepared in the same way as PAM except DI water was replaced with 8 M LiCl solution, and 0.6 wt % BIS was used to make it more elastic. The PAM-LiCl precursor was then pipetted to the PDMS mold with a dimension of 6 mm by 6 mm by 1.5 mm. The PDMS-BP film was placed atop the PDMS mold filled with PAM-LiCl precursor and subjected to UV irradiation (Omicure, 365 nm) at 60 mW/cm^2^. After 4 min, the UV was deactivated, and the mold was gently removed. Subsequently, another PDMS-BP film was applied to the flipped side of the PAM-LiCl and irradiated with UV for an additional 6 min.

### Mechanical tests

Unless otherwise indicated, all gels were washed using tap water to remove the residual unreacted chemicals and dried using air blow before being adhered to the cut leaves. The stress-strain curves were obtained by tensile tests using rectangular strips (10 mm by 35 mm by 2 mm) of both CS/PAM and PAM of different BIS concentrations. To measure interfacial toughness, leaves were cut from the plants and fixed on the glass slides to use as the substrates. Gels with widths of 10 mm were prepared and adhered to the surfaces of the leaves by pressing with 500-Pa pressure using a weight for 2 min. Scotch tape (3M) was applied as a stiff backing layer for the gel. For interfacial toughness measurements under surface water exposure, external water was applied to the gel surface before attaching the stiff backing tape. This ensures consistent testing conditions, as the tape is impermeable to water. Ninety-degree peeling tests were carried out using a mechanical testing machine equipped with a 90° angle peel fixture (Instron Corp.; 100-N load-cell). All tests were conducted with a constant peeling speed of 50 mm/min. Interfacial toughness was determined by dividing the plateau force by the width of the gel sample.

To measure shear strength, gels were prepared with a dimension of 10 mm by 12 mm by 2 mm and sandwiched by the cut leaves. Scotch tape (3M) was applied as a stiff backing layer for the cut leaves. The lap shear test was conducted using a mechanical testing machine (Instron Corp.; 100-N load cell). All tests were conducted with a constant tensile speed of 50 mm/min. Shear strength was determined by dividing the maximum force by the adhesion area (10 mm by 12 mm). Adhesion performance was compared with three commercial tapes (3M magic tape, 3M delicate surface tape, and EZlifego double-sided tape) using 90° peeling tests on benth and cowpea leaves. The release liner on the EZlifego tape was retained during testing to serve as a stiff backing and ensure consistent measurements.

### Chlorophyll content measurement

SPAD-502 value was obtained from the chlorophyll meter (2900P, Spectrum Technologies Inc.). Biopsied gel discs (0.5 cm in diameter) were applied to the leaf surfaces in experimental groups, while no treatment was applied to control groups. Plants were watered after the measurement on day 4. The chlorophyll meter was calibrated by the reading checker before use. SPAD value of each leaf was averaged from five readings at different spots. The SPAD value was obtained from three leaves for each measurement.

### FTIR spectroscope characterization

FTIR spectroscope (Nicolet iS50, Thermo Fisher Scientific) was used to characterize the chemical composition of the leaves and gels using a transmission with a germanium-attenuated total reflectance crystal.

### Gel adhesion under simulated rainfall

Rain mimicking including drizzle and violent rainfall was conducted on plants to further examine the stability of gel adhesion on plants under different outdoor conditions. According to MANOBS, rainfall events are classified on the basis of rainfall depth and intensity. To simulate drizzle, a spray bottle was used to replicate the small droplet size characteristic of light rain. For simulating heavy rainfall, a standard showerhead was used to deliver a high-volume water flow resembling intense precipitation. Rainfall depth is calculated from rainwater volume collected over a specific period and measured as the height of water, and rainfall intensity is determined by the total amount of rain per duration. In our experimental setup, rainfall depth corresponds to the total amount of rain over the shower area, while the rainfall intensity is considered rainfall depth divided by period. Interfacial toughness of adhesive gels on both benth and cowpea was measured after simulated drizzle and violent rain conditions using the same 90° peeling procedure described above.

### Cryosectioning of leaves

Benth leaves treated with QD-loaded adhesive/nonadhesive gels and QD liquid solution were cryosectioned to analyze the QD delivery in leaf tissue under fluorescent microscopy. Benth leaves were harvested after 4 hours of treatment. The surfaces of the benth leaves were flushed with DI water to remove any residual QDs on the surfaces and wiped using Kimwipes to remove surface water. The benth leaves were then embedded in Tissue-Tek O.C.T. Compound and frozen in liquid nitrogen. Samples were stored in a −80°C freezer for further use. A Leica CM1860 cryostat (Leica Microsystems) was used to obtain a 50-μm section of the leaves.

### Fluorescent and real-time imaging

To quantify the delivery efficiency of QDs using adhesive gels, nonadhesive gels, and liquid solution, fluorescent imaging was conducted on cryosectioned leaf samples using Nikon Eclipse Ni fluorescence microscope equipped with a Nikon DS Qi2 camera at ×10 magnification. Pixel intensity of QD signal was captured at a fixed exposure time of 400 ms under DAPI filter and then analyzed using the mean gray value in ImageJ software. To calculate the diffusion speed of the QDs from the gels or liquid solution to plant tissue, fluorescence images were collected using the EVOS Imaging System (Thermo Fisher Scientific). A benth leaf from an intact live plant was mounted using 3M tape on the microscope stage. The QD-loaded gels, as described above, or 400 μl of QD solution (1 mg/ml), were applied to benth leaves. The built-in blue light tube (447 to 460 nm) in the EVOS Imaging System was used to excite the QD. Images were collected automatically every 20 min for a total period of 4 hours. The collected images were used to calculate the diffusion speed analyzed by Fiji (ImageJ), where the diffusion speed is calculated on the basis of the diffusion distance divided by time.

### Cultivation of benth and cowpea

Benth seeds were germinated in Pro-Mix BX soil (Greenhouse Megastore) in 3–1/4″ square pots (Greenhouse Megastore) and grown for 4 weeks in an A1000 chamber (Conviron) at 25/22°C (day/night cycle), 70% RH, and ~100,000 lux (16-hour photoperiod). Fertilizer (JR Peters, catalog no. 77860) was added during irrigation at a concentration of 0.5 g/liter after seeding and then every 2 weeks of cultivation. Cowpea (Vigna unguiculate no. 5, Morgan County Seeds) plants were grown under the same conditions for 1 week but without fertilizer.

### Antibiotic tolerance

To test the OTC tolerance of benth plants, gels were incubated in OTC at a concentration of 1.25 or 2.5 mg/ml overnight. OTC-loaded gels were then briefly rinsed with DI water and carefully attached to the abaxial leaf surface of benth leaves. Plants were incubated as described above and imaged 24 hours after attaching the adhesive gels.

### Leaf infection assays

Transgenic *A. tumefaciens* containing the vector pJL-TRBO-G (Addgene, plasmid #80083) were grown in 500-ml glass flasks containing 50 ml of LB medium [tryptone (10 g/liter), yeast extract (5 g/liter), and NaCl (10 g/liter) (pH 7.0)], supplemented with rifampicin (25 mg/liter) and gentamycin (50 mg/liter) and kanamycin (50 mg/liter). Cultures were incubated at 28°C for ~24 hours at 160 rpm. After reaching an optical density at 600 nm (OD_600 nm_) of ~5.0, *A. tumefaciens* cultures were harvested by centrifugation at 3200*g* for 10 min at room temperature. Supernatants were discarded, and cell pellets were resuspended in infiltration buffer [30 mM MES buffer, Murashige and Skoog major and minor salts (0.5 g/liter), and 200 μM acetosyringone (pH 5.6)], followed by induction at 22°C for 1 hour at 80 rpm.

For leaf infection assays, agrobacterium suspensions were adjusted to a final OD_600 nm_ of 0.1, using the same infiltration buffer. The cell suspension (50 μl) was then syringe infiltrated through the abaxial surface of benth leaves 0, 4, or 16 hours after attaching biopsied gel discs (0.8 cm in diameter) containing OTC (1.25 mg/ml). Gels were attached to the abaxial leaf surface as described above; per plant, two gels were attached on separate leaves. Infection events were visualized 2 days postinfiltration through the expression of GFP using two UV lamps (Analytic Jena) for excitation. Photographs obtained under UV lamps were analyzed using ImageJ for a quantitative comparison of GFP intensities in benth leaves.

### Signal module fabrication

The circuit module we fabricated consists of a bridge rectifier, an Arduino Nano, and a step-down voltage regulator. The rectifier converts the TENG signal to dc values so the Arduino can read the signal on its analog port. Once the Arduino detects a voltage greater than 2.5 V, it outputs a high pulse on a digital port for 0.25 s. The digital output goes into the step-down voltage regulator, which converts the output of the digital port to 3.2 V for plant actuation.

### Electrical performance of gels

To ensure a defined measurement area, PAM-LiCl, CS/PAM, and CS/PAM-CaCl_2_ gels were diced into pieces with dimensions of 5 mm by 5 mm. Gel samples were stored in a temperature-humidity chamber at room temperature and 80% RH to prevent dehydration. The impedance and series resistance as a function of electrode frequency were measured using an impedance analyzer (IM3570, HIOKI). The electrical conductivity (σ) was calculated from the series resistance (*R*_s_) according to the following equation

$$\sigma =L/R_{s}A$$

where σ is the conductivity, *R*_s_ is the series resistance, *L* is the thickness of the gel sample, and *A* is the electrode area.

### Measurement of triboelectric performance

Electrical measurements of the TENG are recorded by a sourcemeter (2400, Keithley), an oscilloscope (DPO 2022B, Tektronix, USA), and a customized signal module connected to a customized pushing tester (SnM). Operation conditions of the pushing tester are set under the pressure of 49 kPa, the contact speed of 1 Hz, and the contact distance of 2 mm between the contact material (i.e., Al) and TENG. The contact area remains at 1 cm^2^.
