# Supplementary material for: A strong, reversible, and conformal adhesive gel for diverse plants
Source: Sci Adv. 2026 Apr 24;12(17):eadz6379. doi: 10.1126/sciadv.adz6379 (PMC13108563; doi:10.1126/sciadv.adz6379)
Supplement: Supplementary file 1 — Figs. S1 to S18 Table S1 Legends for movies S1 to S15 [file sciadv.adz6379_sm.pdf]

Science Advances
AAAS

Supplementary Materials for

A strong, reversible, and conformal adhesive gel for diverse plants

Jiayu Zhao *et al.*

Corresponding author: Nicole F. Steinmetz, nsteinmetz@ucsd.edu; Jinhye Bae, j3bae@ucsd.edu

Sci. Adv. **12**, eadz6379 (2026)
DOI: 10.1126/sciadv.adz6379

The PDF file includes:

Figs. S1 to S18
Table S1
Legends for movies S1 to S15

Other Supplementary Material for this manuscript includes the following:

Movies S1 to S15

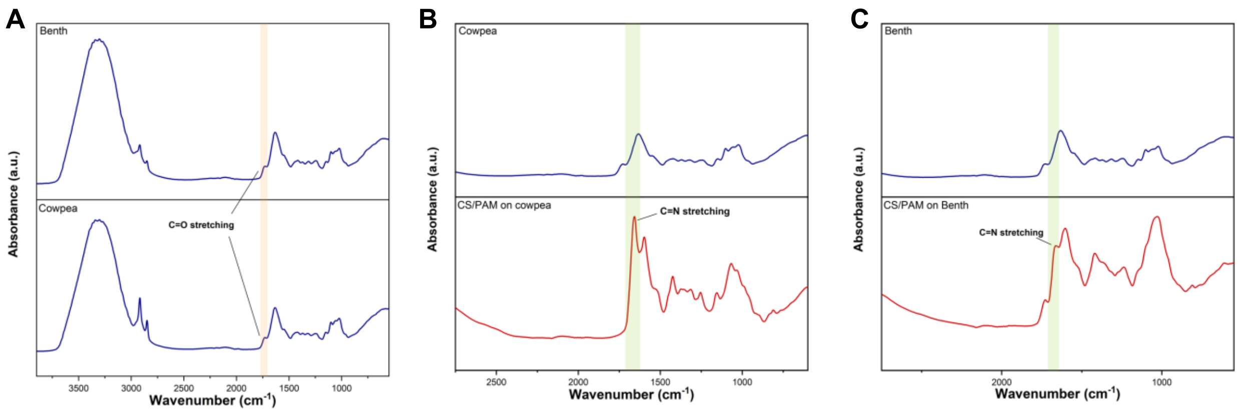

Fig. S1. Fourier transform infrared spectroscopy analysis of pristine and CS/PAM-coated plant leaves. (**A**) Pristine benth and cowpea leaves. (**B**) Pristine cowpea leaf and CS/PAM attached on cowpea leaf after 12h. (**C**) Pristine benth leaf and CS/PAM attached on benth leaf after 12 hours. C=O stretching at 1727 cm^{-1}; C=N stretching at 1664 cm^{-1}.

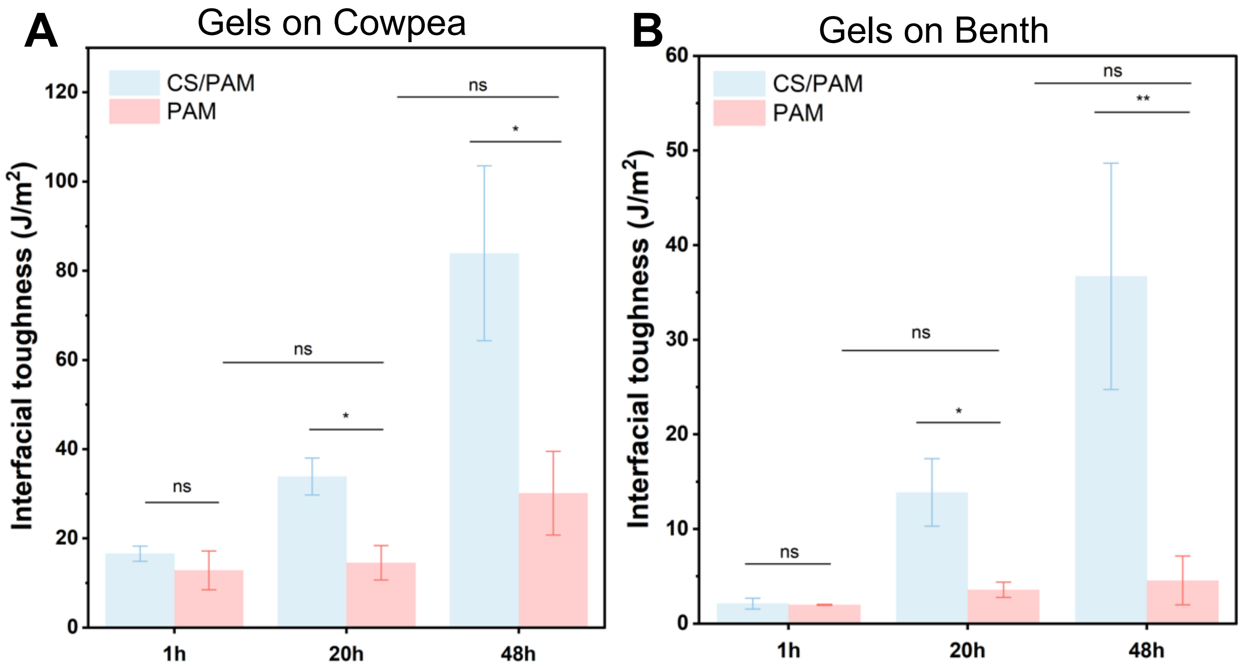

Fig. S2. Time-dependent interfacial toughness of gels on plant leaves. The time-dependent interfacial toughness between (**A**) gels and cowpea and (**B**) gels and benth, respectively. The gels under-tested are CS/PAM and PAM. The error bars represent the SD. Statistical analysis was performed by paired t-test. *$P < 0.05$, **$P < 0.01$, ****$P < 0.0001$, ns = not significant.

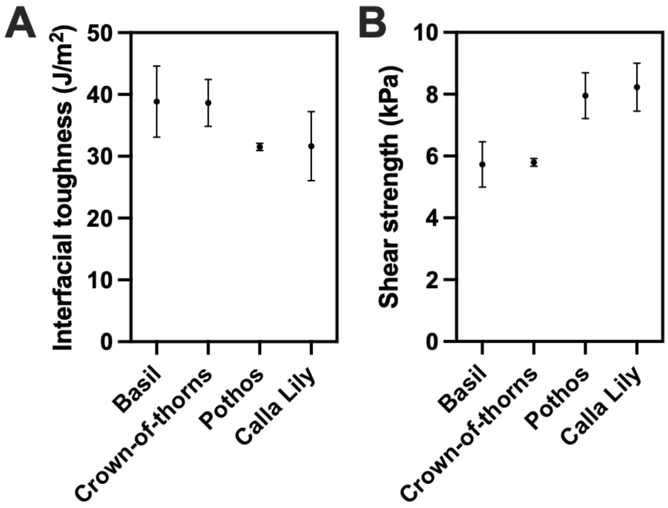

Fig. S3. Adhesion performance of adhesive gels on diverse plant leaves. (**A**) Interfacial toughness and (**B**) shear strength of the optimized CS/PAM gel (CS/PAM with 0.06 wt% BIS) after applied for 1 hour on basil, crown-of-thorns, pothos, and calla lily leave adaxial surfaces, respectively. The error bars represent the SD.

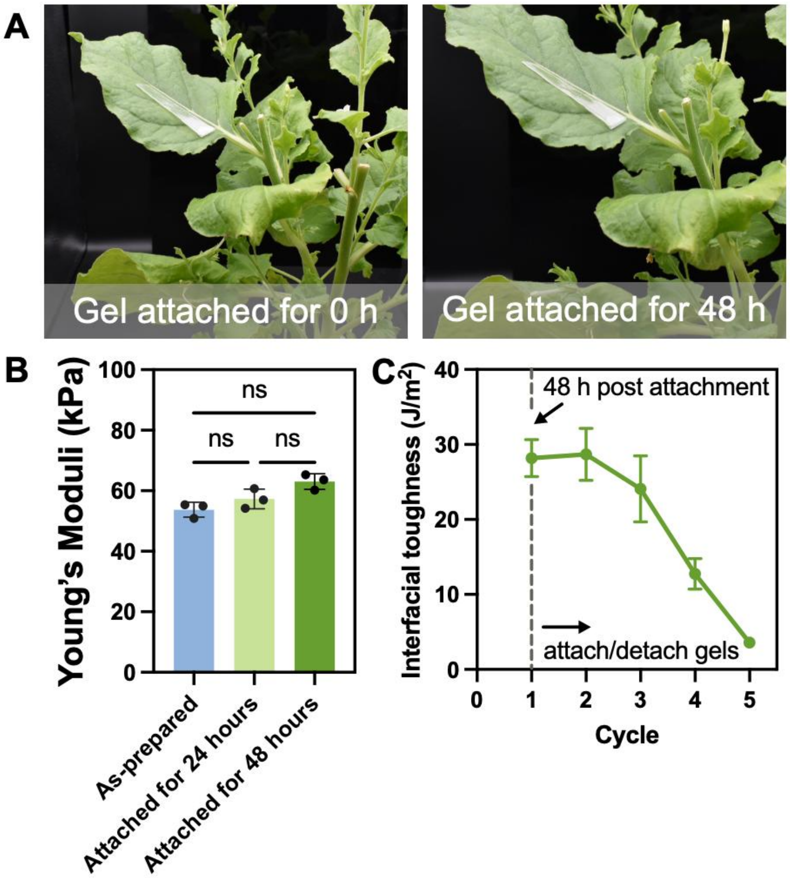

Fig. S4. Durability and mechanical properties of adhesive gels on plant leaves. (**A**) Photographs of gel attached to benth for 0 and 48 hours. (**B**)Young's modulus of the as-prepared gels and gels attached to the leaf for 24 hours and 48 hours. (**C**) Repeatability of gel adhesion on plants. Interfacial toughness was measured on freshly cut leaves taken from the same plant immediately after a 48-hour gel attachment. The error bars represent the SD. Statistical analysis was performed by one-way ANOVA with a post-hoc Tukey's test. *P < 0.05, **P < 0.01, ****P < 0.0001, ns = not significant.

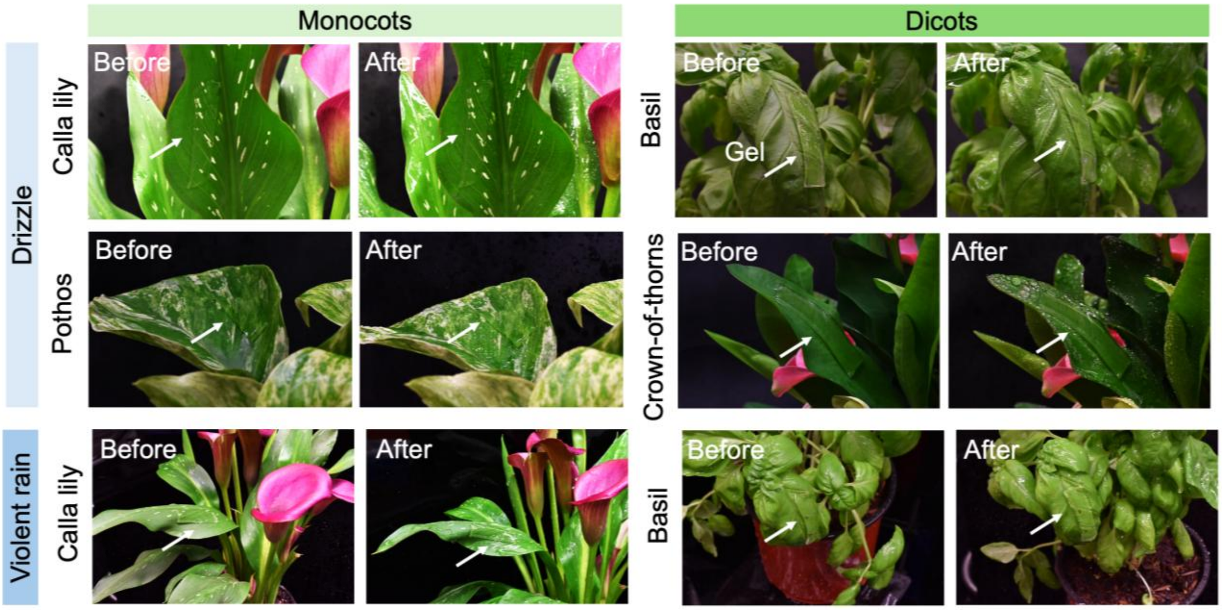

Fig. S5. Stability of gel attachment under simulated rain conditions. Stable gel attachment on various plant surfaces in 1-minute drizzle (produced by spray bottle) and 20-minute violent rainfalls (generated by showerhead). Arrows indicate the location of the gel.

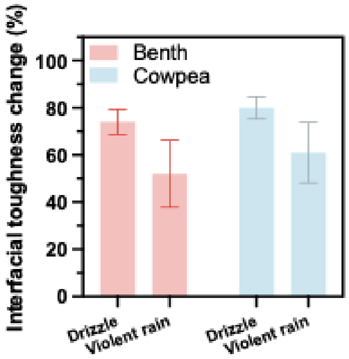

Fig. S6. Interfacial toughness of CS/PAM on plant leaves. Measured before and after exposure to simulated drizzle and violent rain conditions. The error bars represent the SD.

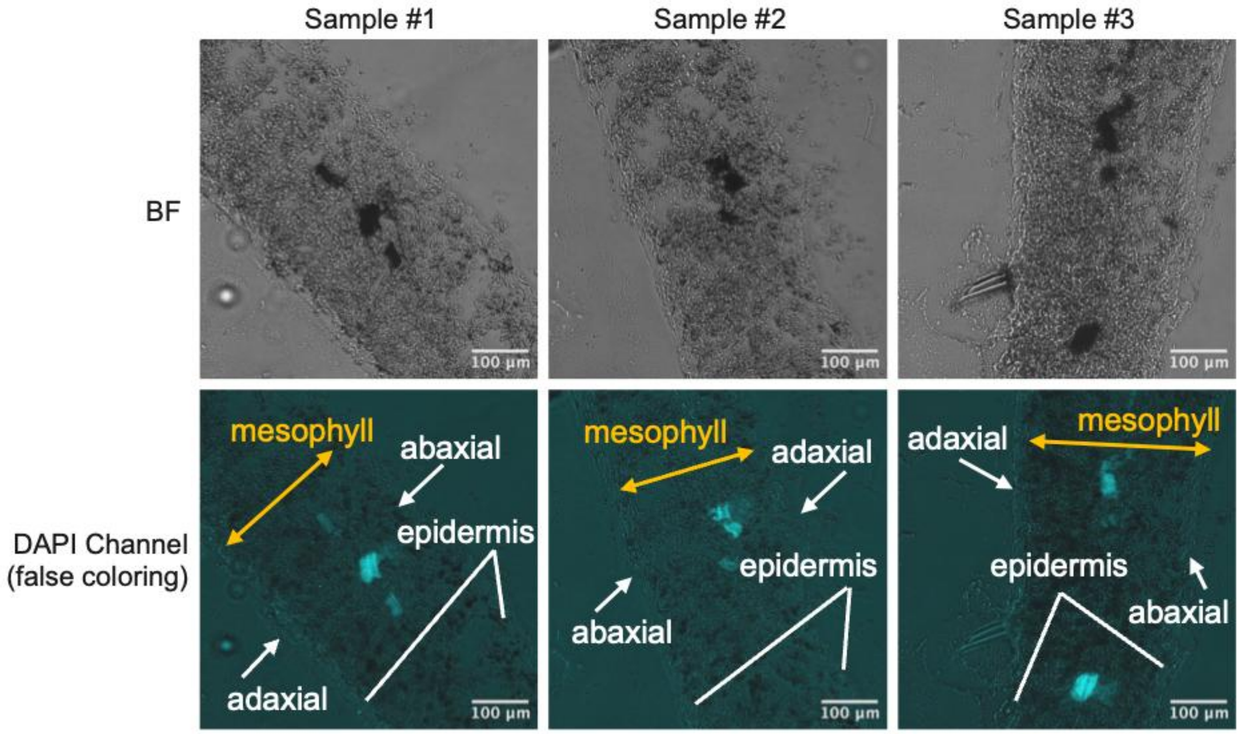

Fig. S7. Fluorescent microscope images of QDs diffusion in cryosectioned benth leaves after adhesive gel treatment. Benth leaves were treated with QD-loaded CS/PAM (0.06 wt% BIS) for 4 hours. Images were taken under bright field (BF) and 4′,6-diamidine-2′-phenylindole (DAPI) channel.

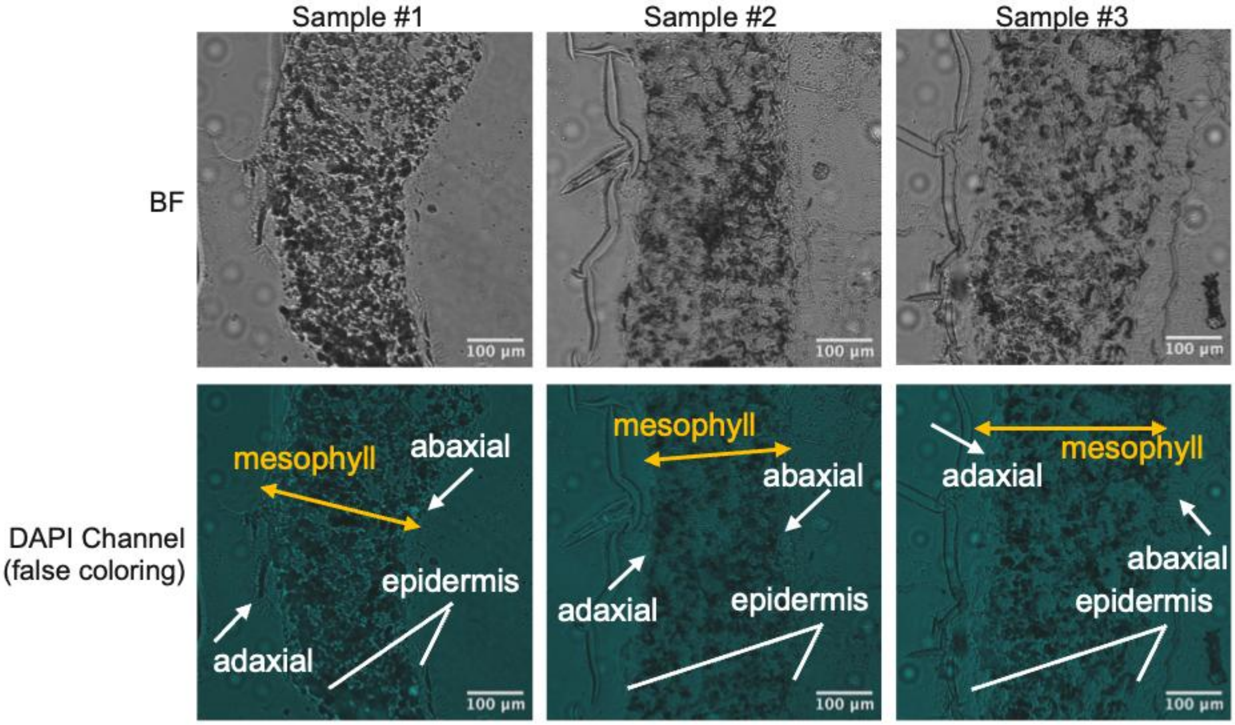

Fig. S8. Fluorescent microscope images of QDs diffusion in cryosectioned benth leaves after non-adhesive gel treatment. Benth leaves were treated with QD-loaded CS/PAM (0.6 wt% BIS) for 4 hours.

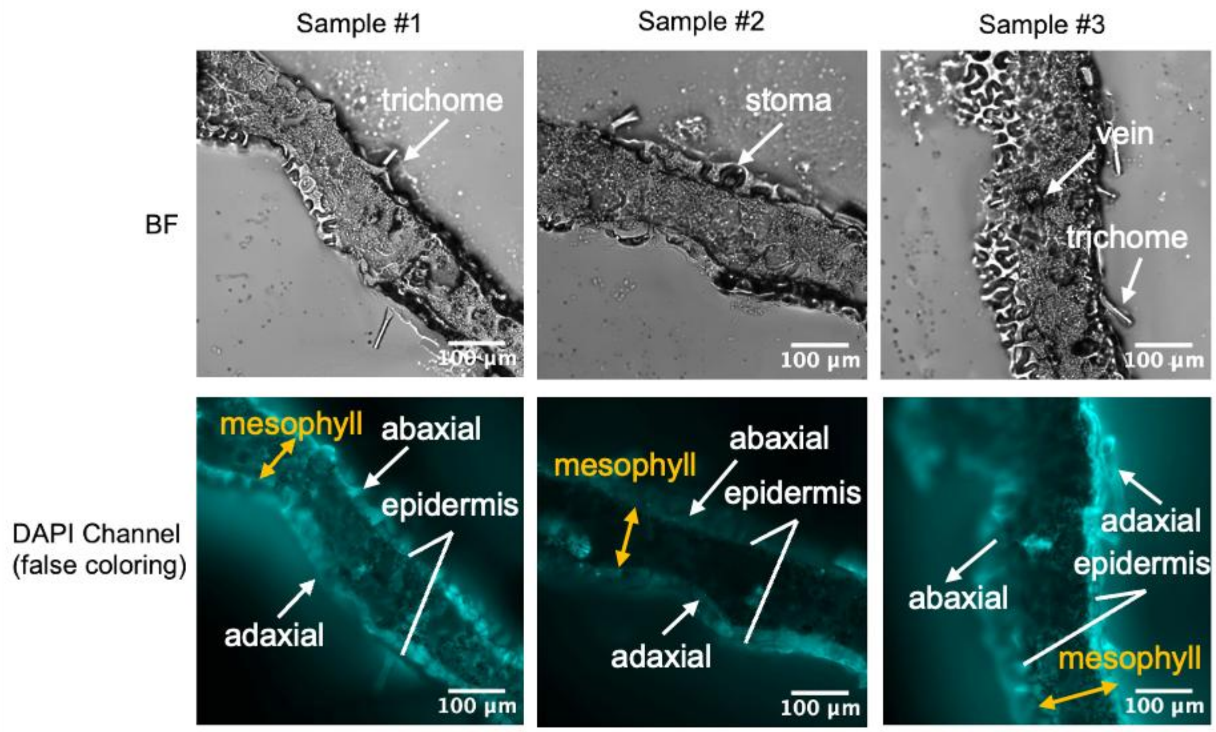

Fig. S9. Fluorescent microscope images of QDs diffusion in cryosectioned benth leaves after QDs liquid solution treatment. Benth leaves were treated with QDs liquid solution (1 mg/mL) and waited for 4 hours before washing and cryosectioning.

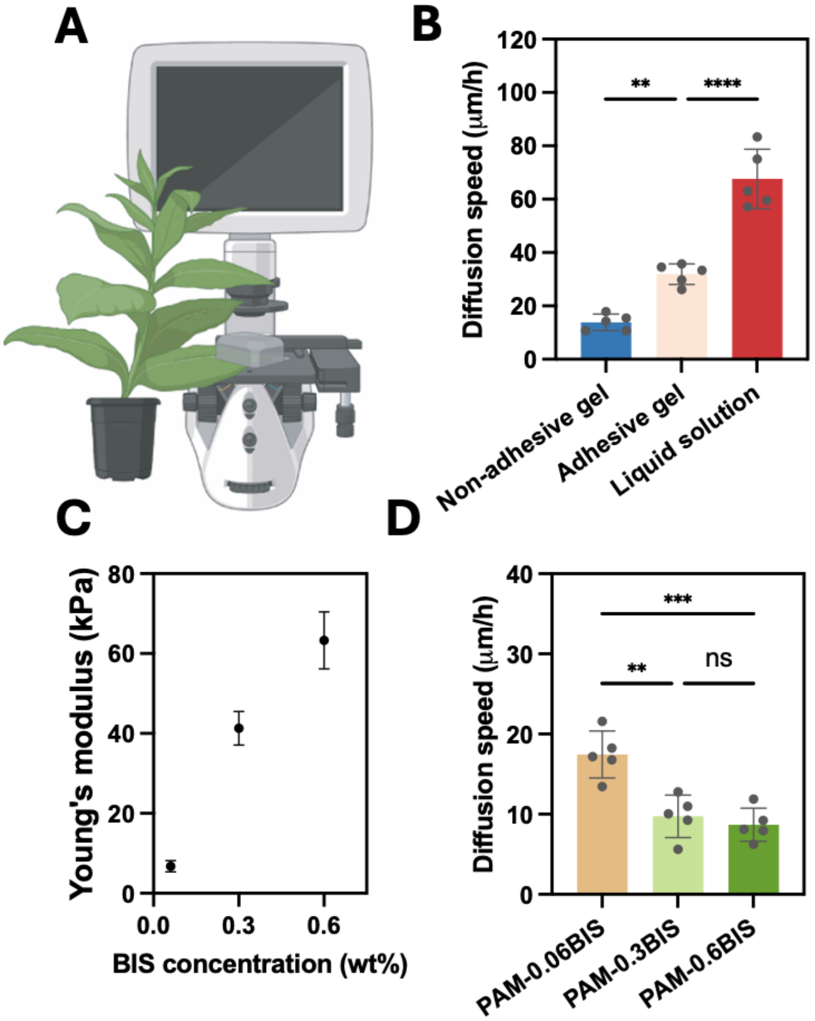

Fig. S10. Real-time imaging and analysis of QDs diffusion in plant leaves. (**A**) Schematic of the real-time imaging of QDs diffusion in benth, where the benth was mounted on the microscope stage with QDs-loaded gels or QDs liquid solution on benth adaxial surface. Images were taken at a 20-min interval over 4 hours. (**B**) The corresponding QDs diffusion speed in the leaf calculated based on the real-time imaging results. (**C**) Young's moduli of PAM gels with BIS concentrations of 0.06 wt%, 0.3 wt%, and 0.6 wt%. (**D**) QDs diffusion speed of PAM gels with different BIS concentrations in the leaf calculated based on the real-time imaging results. The error bars represent the SD. Statistical analysis was performed by one-way ANOVA with a post-hoc Tukey's test. *$P < 0.05$, **$P < 0.01$, ****$P < 0.0001$, ns = not significant. Fig. S10A schematic was created with BioRender. Guan, Z. (2026) (https://BioRender.com/w13b896)

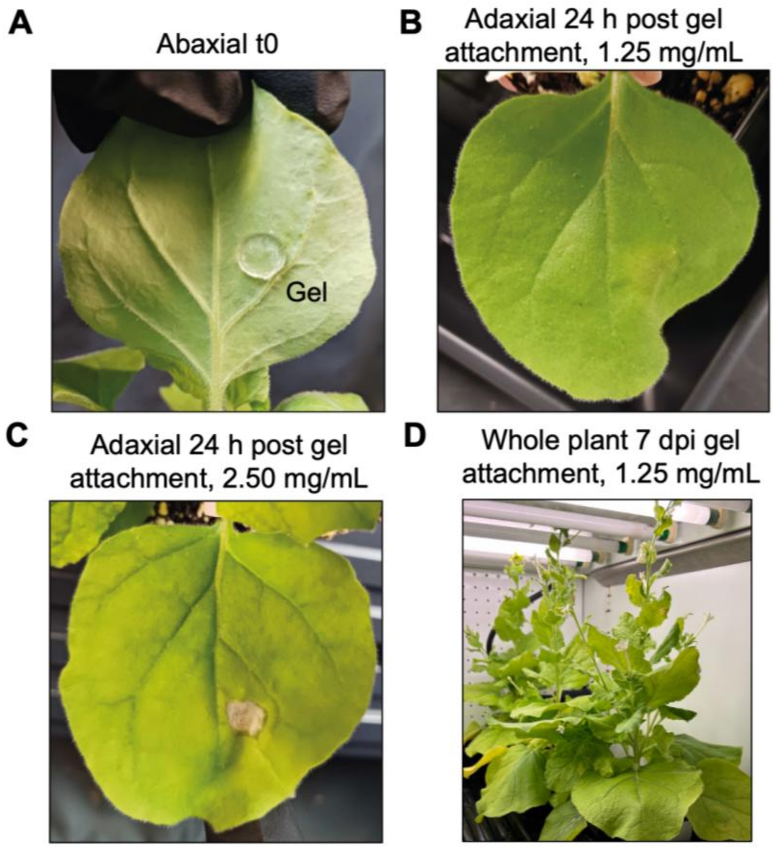

Fig. S11. Tolerated antibiotic concentrations. Images of leaves after (**A**) 0 and (**B, C**) 24 hours of gel attachment. Adhesive gels immersed in 1.25 or 2.5 mg/mL oxytetracycline (OTC) solutions were attached to the abaxial leaf surface of benth plants (n=3). Gels were attached on the abaxial leaf surface, because the thicker cuticle on the adaxial surface interferes with antibiotic uptake. High (2.5 mg/mL) OTC concentrations induced necrosis in leaf tissue that was in direct contact with the adhesive gel. (**D**) Image of the whole plant after 7 days of gel attachment (1.25 mg/mL).

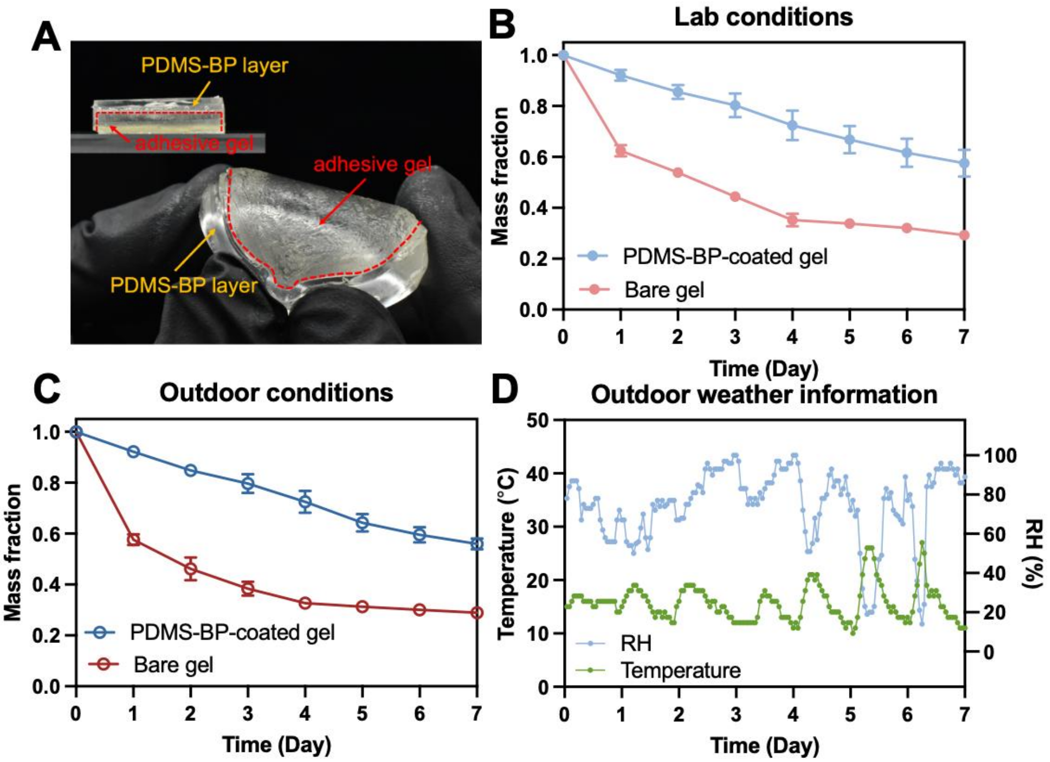

Fig. S12. Dehydration of adhesive gels. (**A**) Photograph of the gel with a PDMS-BP layer, showing a seamless interface between the PDMS-BP layer and the adhesive gel. The inset photograph shows the side-view of the PDMS-BP layer-coated gel. Mass fraction of bare adhesive gel and PDMS-BP layer coated gel under (**B**) lab conditions and (**C**) outdoor conditions over 7 days. (**D**) Hourly local weather information during mass fraction recording, collected from https://www.timeanddate.com/weather/. The error bars represent the SD.

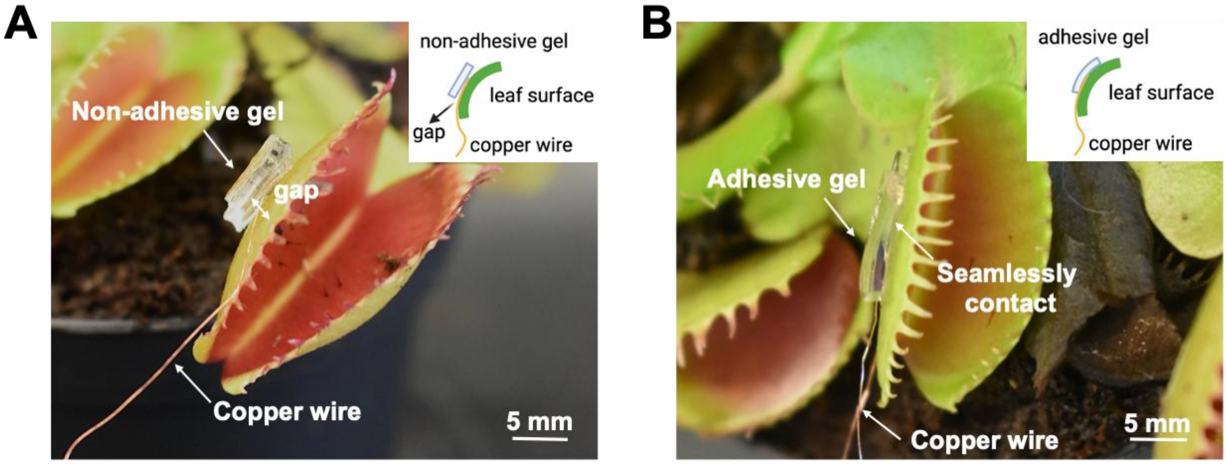

Fig. S13. Comparison of non-adhesive and adhesive gel interfaces on a curved plant surface. (**A**) Photographs and inset scheme showing the non-adhesive gel failed to securely hold the copper wire due to the gap created between the stiff gel and curved surface of the Venus flytrap. (**B**) Photographs and inset scheme showing the adhesive gel holding a copper wire by forming seamless contact with the curved surface.

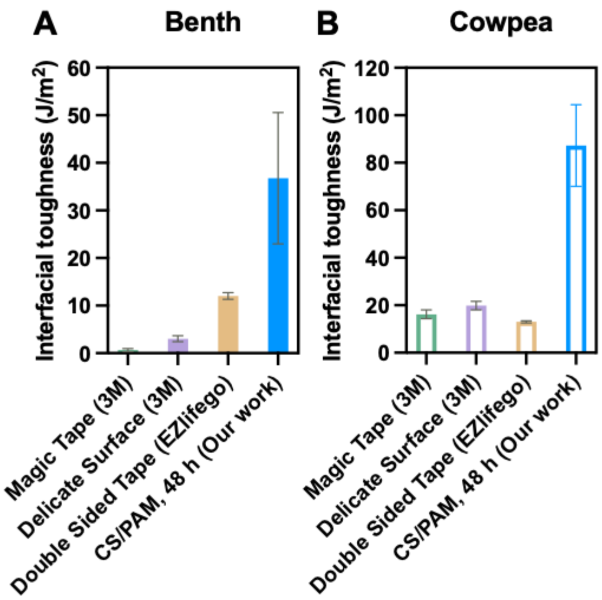

Fig. S14. Interfacial toughness of commercial adhesives and CS/PAM on plant leaves. Measured on (**A**) benth and (**B**) cowpea, respectively. The error bars represent the SD.

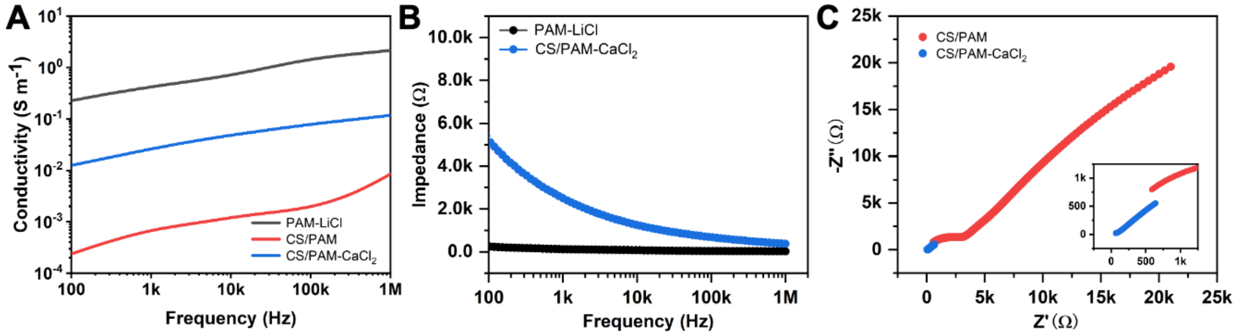

Fig. S15. Electrical properties of gels. (**A**) Electrical conductivity of PAM-LiCl, CS/PAM, and CS/PAM-$CaCl_2$ gels as a function of AC frequency. (**B**) Impedance of PAM-LiCl and CS/PAM-$CaCl_2$ gels as a function of AC frequency. (**C**) Nyquist plot of CS/PAM and CS/PAM-$CaCl_2$ gels.

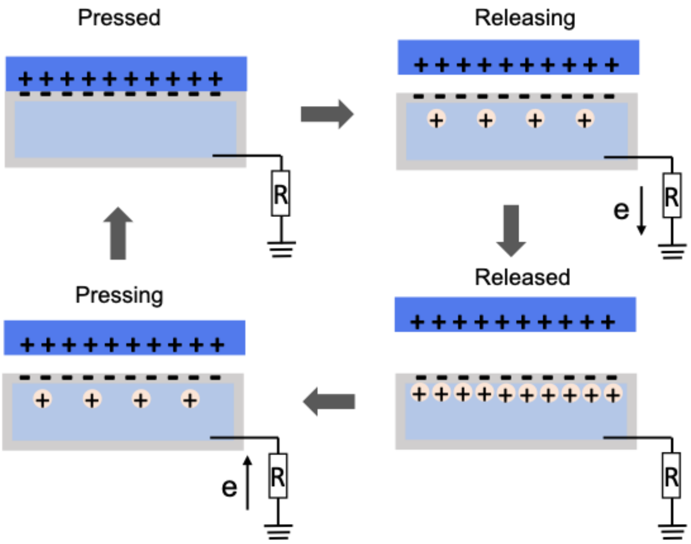

Fig. S16. Working mechanism of the TENG based on contact–separation motion. Schematic illustrating the operating principle of the TENG during contact and separation cycles.

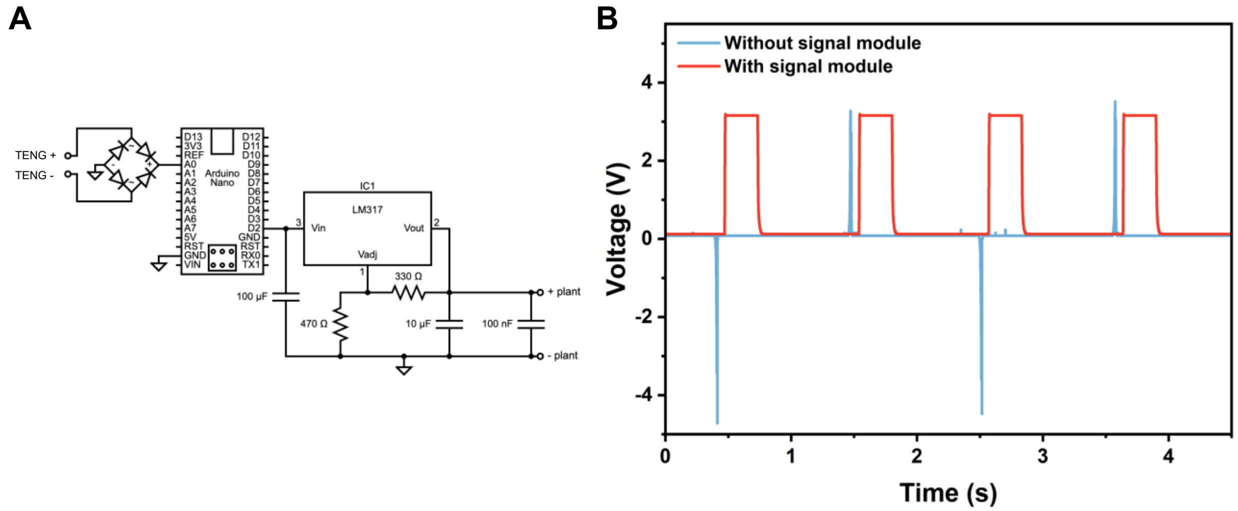

Fig. S17. Signal generation and measurement of the wearable TENG. (**A**) Circuit diagram of the signal module. (**B**) Voltage signals of the wearable TENG generated without (blue) and with (red) the signal module.

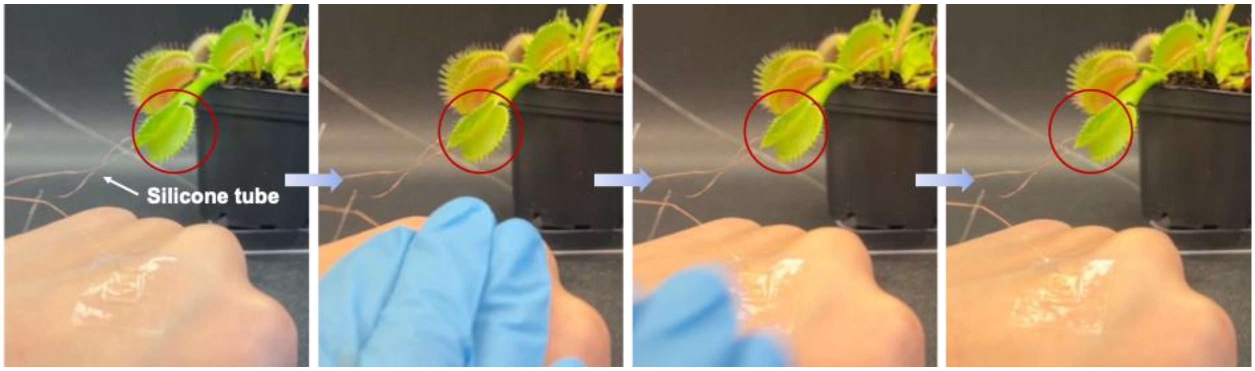

Fig. S18. Non-conductive connection between wearable TENG and Venus flytrap. Series of photographs showing that the Venus flytrap cannot close its trap upon pressing the wearable TENG as the conductive copper wire connecting the signal module to the adhesive gel on the Venus flytrap is replaced with the non-conductive silicone tube.

Table S1. Summary of adhesion performance and applications of non-invasive plant adhesives

Publications	Adhesion mode	Interfacial toughness (J/m^2)	Shear strength (kPa)	Reversibility (Y/N/N/A)	Applications
**This work**	**Pre-formed**	**87.2 (cowpea)<br>36.7 (benth)**	**10.1 (cowpea)<br>3.3 (benth)**	**Y**	**Antibiotic delivery<br>Human-plant interaction**
Skin-inspired all-natural biogel for bioadhesive interface (*34*)	*In situ* gelated	~28 (tomato plant)	N/A	N/A	Plant electrophysiology monitoring
All-organic transparent plant e-skin for noninvasive phenotyping (*35*)	Pre-formed	N/A	0.20-0.38 N*<br>(various plants)	N	Plant electrophysiology monitoring
A morphable ionic electrode based on thermogel for non-invasive hairy plant electrophysiology (*19*)	*In situ* gelated	N/A	0.15 (benth)	N/A	Plant electrophysiology monitoring
Cohabiting plant-wearable sensor in situ monitors water transport in plant (*36*)	Pre-formed	N/A	N/A	Y	Plant electrophysiology monitoring
Switchable adhesion of hydrogels to plant and animal tissues (*37*)	Electroadhesion	N/A	4-20 (inner side of strawberry); failed on plant leaves	Y	Biohybrid of gel and plant tissues
An on-demand plant-based actuator created using conformable electrodes (*38*)	Pre-formed	15.6 (Venus flytrap)	N/A	N/A	Human-plant interaction

*Only adhesion force was mentioned in the lap shear test, and we could not normalize this value to shear strength as the contact area is missing. For comparison, the adhesion forces of our adhesive gel on leaf adaxial surfaces of all 6 plant species are larger than 0.4 N.

Movie S1. Non-adhesive gel (CS/PAM with 0.6 wt% BIS) applied to a benth leaf surface subjected to airflow.
Movie S2. Adhesive gel (CS/PAM with 0.06 wt% BIS) applied to a benth leaf surface subjected to airflow.
Movie S3. Adhesive gel applied to a basil exposed to drizzle mimicked by mist from a spray bottle for 1 minute.
Movie S4. Adhesive gel applied to a crown-of-thorns exposed to drizzle mimicked by mist from a spray bottle for 1 minute.
Movie S5. Adhesive gel applied to a pothos exposed to drizzle mimicked by mist from a spray bottle for 1 minute.
Movie S6. Adhesive gel applied to a calla lily exposed to drizzle mimicked by mist from a spray bottle for 1 minute.
Movie S7. Adhesive gel applied to a basil exposed to violent rain mimicked by flow from a showerhead for 20 minutes.
Movie S8. Adhesive gel applied to a calla lily exposed to violent rain mimicked by flow from a showerhead for 20 minutes.
Movie S9. QDs diffusion in 4 hours in benth attached with QDs-loaded adhesive gel.
Movie S10. QDs diffusion in 4 hours in benth attached with QDs-loaded non-adhesive gel.
Movie S11. QDs diffusion in 4 hours in benth treated with QDs liquid solution.
Movie S12. Non-adhesive gel applied to a Venus flytrap lobe fails to hold a copper wire under disturbance.
Movie S13. Adhesive gel applied to a Venus flytrap lobe securely holds a copper wire under disturbance.
Movie S14. TENG-actuated closure of a Venus flytrap facilitated by adhesive gel.
Movie S15. Same setup as Movie S14, but the conductive copper wire is replaced with a non-conductive silicone tube, demonstrating that the Venus flytrap actuation is driven by the voltage generated by the TENG, rather than subtle mechanical disturbances caused by pressing the TENG device.
